# Examination of hydrogen cross-feeders using a colonic microbiota model

**DOI:** 10.1186/s12859-020-03923-6

**Published:** 2021-01-06

**Authors:** Nick W. Smith, Paul R. Shorten, Eric Altermann, Nicole C. Roy, Warren C. McNabb

**Affiliations:** 1 School of Food and Advanced Technology, Massey University, Palmerston North, New Zealand; 2 Riddet Institute, Massey University, Private Bag 11222, Palmerston North, 4442 New Zealand; 3 AgResearch, Ruakura Research Centre, Private Bag 3123, Hamilton, 3240 New Zealand; 4 AgResearch, Grasslands Research Centre, Private Bag 11008, Palmerston North, 4442 New Zealand; 5 High-Value Nutrition National Science Challenge, Auckland, New Zealand; 6 Department of Human Nutrition, University of Otago, Dunedin, New Zealand; 7 Liggins Institute, The University of Auckland, Auckland, New Zealand

**Keywords:** microPop, Community modelling, Methane, Hydrogen sulphide, Microbiome

## Abstract

**Background:**

Hydrogen cross-feeding microbes form a functionally important subset of the human colonic microbiota. The three major hydrogenotrophic functional groups of the colon: sulphate-reducing bacteria (SRB), methanogens and reductive acetogens, have been linked to wide ranging impacts on host physiology, health and wellbeing.

**Results:**

An existing mathematical model for microbial community growth and metabolism was combined with models for each of the three hydrogenotrophic functional groups. The model was further developed for application to the colonic environment via inclusion of responsive pH, host metabolite absorption and the inclusion of host mucins. Predictions of the model, using two existing metabolic parameter sets, were compared to experimental faecal culture datasets. Model accuracy varied between experiments and measured variables and was most successful in predicting the growth of high relative abundance functional groups, such as the Bacteroides, and short chain fatty acid (SCFA) production. Two versions of the colonic model were developed: one representing the colon with sequential compartments and one utilising a continuous spatial representation. When applied to the colonic environment, the model predicted pH dynamics within the ranges measured in vivo and SCFA ratios comparable to those in the literature. The continuous version of the model simulated relative abundances of microbial functional groups comparable to measured values, but predictions were sensitive to the metabolic parameter values used for each functional group. Sulphate availability was found to strongly influence hydrogenotroph activity in the continuous version of the model, correlating positively with SRB and sulphide concentration and negatively with methanogen concentration, but had no effect in the compartmentalised model version.

**Conclusions:**

Although the model predictions compared well to only some experimental measurements, the important features of the colon environment included make it a novel and useful contribution to modelling the colonic microbiota.

## Background

The microbial population of the human colon has wide-ranging effects on host nutrition and health. These effects include providing important metabolites that cannot be synthesised by the host, modulation of immune functions, and roles in diseases both of the colon and more distant regions of the body (see review by Nicolas and Chang [1]). Efforts to study the colonic microbiota have included observational and interventional studies, coupled with in vitro*,* animal, and computational models. This last technique, although dependent on experimental data for model validation, has appeal as a fast, cheap and high-throughput method, leading to the creation of several mathematical models predicting colonic microbial dynamics in recent years (e.g. [2, 3]). Most of these models focus on the dynamics of the dominant taxa found in the colon and their metabolites, although the importance of other, less abundant microbes is present in the experimental literature. One group of microbes that has largely escaped study in colonic microbiome models includes microbes that metabolise hydrogen [4]. Hydrogen is produced through various microbial metabolic pathways involved in the degradation of carbohydrates in the colon, creating a niche for microbes that can cross-feed on hydrogen [5]. These hydrogenotrophs have demonstrated and hypothesised impacts on both the microbiota and the host, including increasing the rate of carbohydrate fermentation of saccharolytic bacteria in vivo, with associated increases in host adiposity [6], and links to negative health outcomes such as Irritable Bowel Syndrome [7], Inflammatory Bowel Disease [8] and colorectal cancer development [9]. The low relative abundance and inconsistencies in hydrogenotroph abundance between individuals makes them challenging to study [4]. Modelling could provide useful insight into the dynamics of these taxa in vivo, contributing to our understanding of these microbes in human health, nutrition and wellbeing.

Recently, an adaptable tool for modelling the metabolism and growth of microbial communities was published, named microPop [10]. This tool was developed as an extension of a previous model for the in vitro growth of the faecal microbiota published by the same group [11]. microPop models the dynamics of microbial communities by assigning metabolic parameter values to a number of microbial functional groups (MFGs) to be representative of the whole community. The authors stated that the model could be adapted for application to the colon and provided suggestions for how this might be achieved.

Here, an adaptation of the model is presented incorporating these suggestions, alongside further alterations and additions, designed to replicate the behaviour of the major functional groups of the human colonic microbiota. The model was compared to three in vitro faecal fermentation data sets, followed by simulations of the in vivo environment. Adaptations made for the in vivo simulations included the addition of host factors: secreted mucins, host buffering and absorption; as well as microbial adaptations, most notably greater focus on hydrogen cross-feeding microbes and the novel inclusion of sulphate-reducing bacteria (SRB). Finally, various substrate availability scenarios were simulated to give predictions for their effect on the microbiota.

## Results

### Comparison of model predictions to experimental data

microPop constructs and solves a system of ordinary differential equations for any number of MFGs and metabolites, as defined by the user. The base version of microPop (without the additional inclusions to replicate the colon; version 1.5 https://cran.r-project.org/web/packages/microPop/index.html) contains ten colonic MFGs, with a total of 17 metabolic pathways, influencing the concentrations of 20 metabolites. The model utilises Monod kinetics, involving maximum growth rates, half-saturation constants, yield and stoichiometric parameters for each metabolite in each metabolic pathway. The pH and substrate preferences of each MFG are also included, leading to 243 model parameters in total. These parameters were set based on existing knowledge of the MFGs, rather than parameterisation of microPop with faecal culture data. Note that two sets of microbial kinetic parameter values were used in this work: an Alpha set, utilising the original parameter values of Kettle et al. [10]; and a Beta set based on more recent estimates ([12]; see "Methods"). The major differences between the two parameter sets are in the viable and optimal pH range of each MFG, which were altered by as much as 0.95 pH units between parameter sets, and in the maximum growth rates for non-starch polysaccharide (NSP) and resistant starch of all MFGs able to metabolise these substrates. These are the most abundant substrates included in the model, thus substantial changes in model predictions between the two parameter sets were anticipated.

**Fig. 1:**
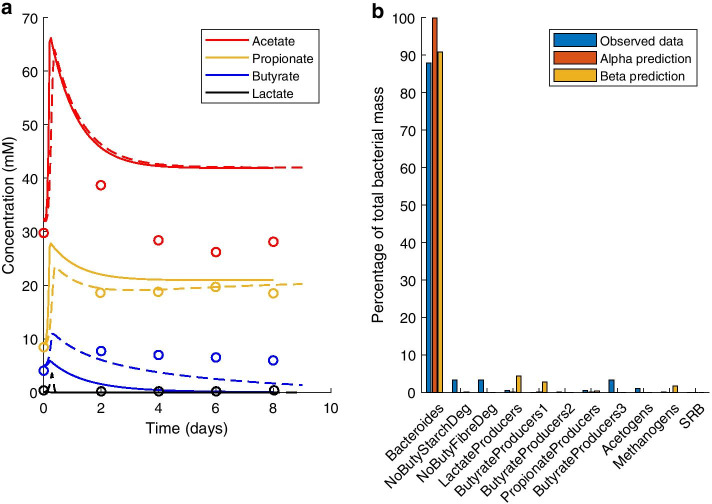
Model prediction compared with experimental data from Walker et al. [13] for continuous culture of a faecal microbial community on a medium containing 0.6% w/v peptide. **a** Measured metabolite concentrations are indicated by the coloured circles, solid lines indicate the model prediction using the Alpha parameter set, and dashed lines indicate the prediction using the Beta parameter set. **b** MFG relative abundance at end of experiment

**Fig. 2:**
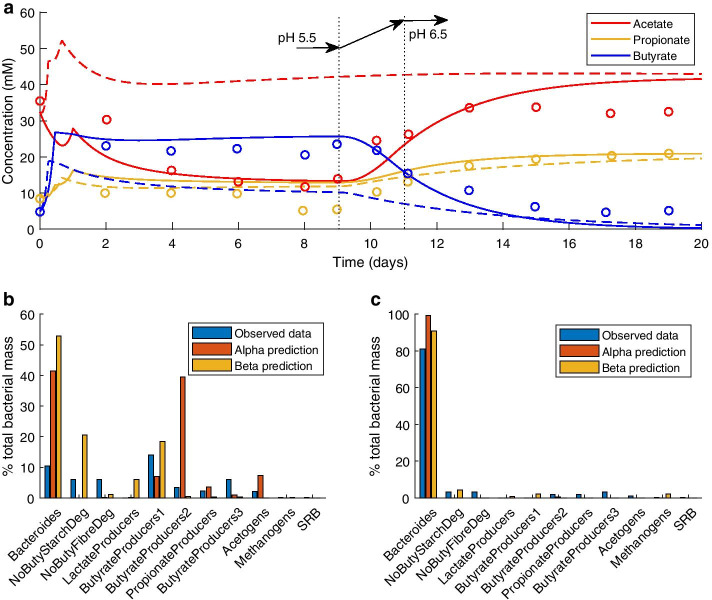
Model prediction compared with experimental data from Walker et al. [13] for continuous culture of a faecal microbial community on medium containing 0.6% w/v peptide. A pH shift from pH 5.5 to pH 6.5 was gradually enacted between days 9 and 11, as shown by the dotted lines in **a**. **a** Measured SCFA concentrations are indicated by coloured circles, solid lines indicate the model prediction using the Alpha parameter set, and dashed lines indicate the prediction using the Beta parameter set. **b** MFG relative abundance before the pH shift (day 9). **c** MFG relative abundance at end of experiment

**Fig. 3:**
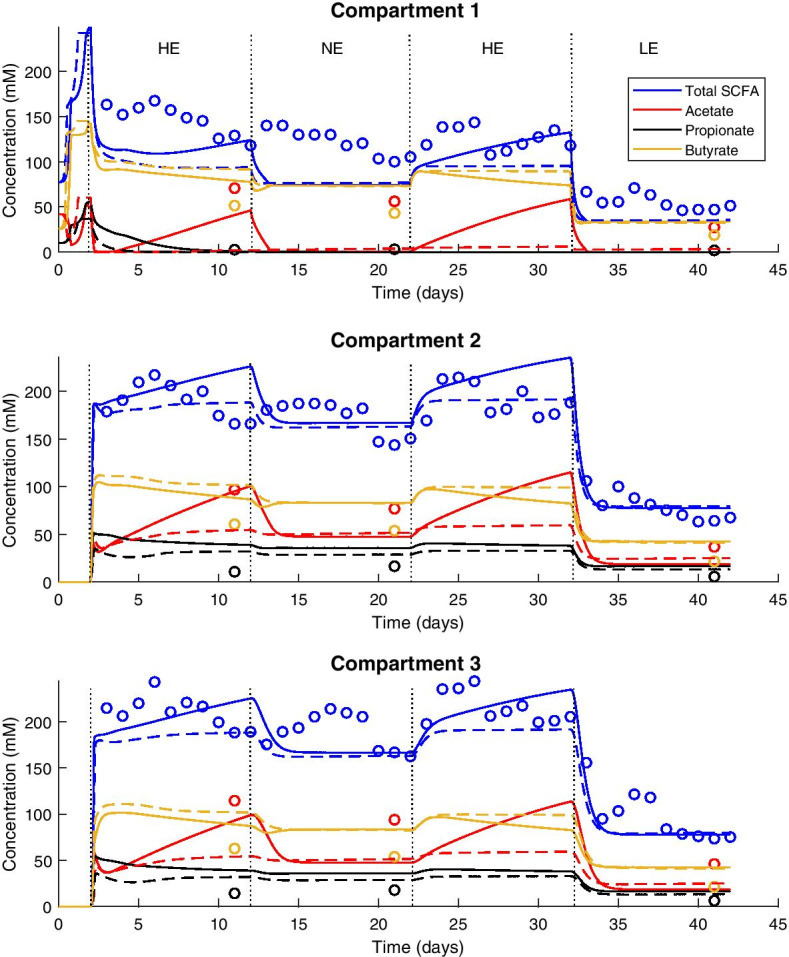
SCFA concentrations in the three fermenter compartments over the course of the 42-day experiment of Payne et al. [14]. This figure pertains to the experiment with faecal material from the obese child, run in batch mode with high-energy (HE) medium for 2 days, then switched to continuous fermentation. During continuous fermentation, the model was run in four 10-day periods with differing media, in the following order: HE, normal-energy (NE), HE, low-energy (LE). Measured data are indicated with coloured circles, solid lines indicate the model prediction using the Alpha parameter set, and dashed lines indicate the prediction using the Beta parameter set. The dotted vertical lines indicate changes between media

To validate that the predictions of microPop were realistic for the colonic MFGs and metabolites modelled, simulated concentrations were compared with experimental data from continuous faecal cultures. The predictions of both parameter sets are shown in each figure in this section. All 20 microPop colonic metabolites were included and the MFGs simulated are listed in Fig. 1b. In total, the model predictions were compared with data from 14 continuous faecal cultures drawn from three previous publications. Figure 1 and Additional file 2: Fig. 1 involve cultures with varied peptide availability. Figure 2 and Additional file 2: Fig. 2 involve cultures with a pH shift during the culture. Figure 3 and Additional Figs. 3, 4 and 5 involve cultures with media changes over time. Additional Figs. 6, 7, 8 and 9 involve cultures from four donors run at either pH 5.5 or pH 6. All simulations were performed with initial conditions and pH values fixed at those used for the experimental work.

Figure 1 shows the microPop prediction for a continuous faecal culture from Walker et al. [13]. This culture was run with 0.6% w/v peptide in the medium; a second experiment was also run with 0.1% w/v peptide (see Additional file 2: Fig. 1). In both cases the pH was fixed at 6.5.

**Fig. 4:**
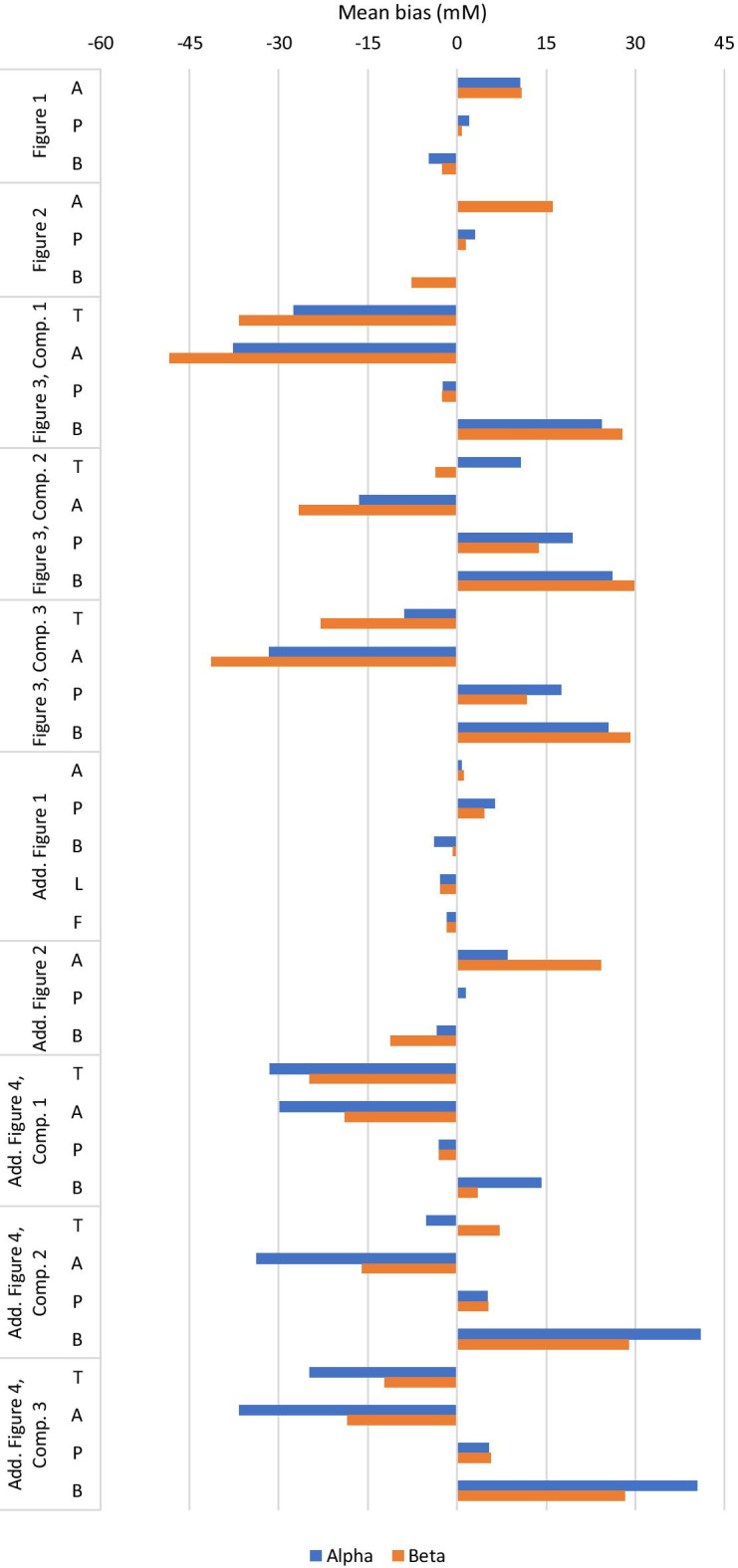
Mean bias of the model fit to experimental data sources for each of the two parameter sets. Neither parameter set gave a better fit to the experimental data than the other across all data sets considered. Comp.: Compartment; Add.: Additional; A: Acetate; P: Propionate; B: Butyrate; T: Total short chain fatty acids; L: Lactate; F: Formate

The model correctly predicted the dominance of the Bacteroides MFG under the high peptide conditions. However, the prediction for short chain fatty acid (SCFA) concentrations were mostly inaccurate (see Fig. 4 and Additional file 2: Table 1). Acetate concentration was overpredicted by at least 15 mM from day 4 of culture onwards using both parameter sets. The model predicted decreasing concentrations of butyrate and lactate, although this was more rapid using the Alpha parameter set than the Beta parameter set, while experimentally both metabolites maintained a non-zero steady state. Contrastingly, propionate concentration was accurately predicted to within 5 mM using the Alpha parameter set and to within 2 mM using the Beta parameter set throughout the experiment.

The model prediction under low peptide conditions (Additional file 2: Fig. 1) for acetate concentration was within 5 mM of the experimental values throughout the experiment using both parameter sets, and for butyrate was within 3 mM using the Beta parameter set. However, the model predicted all other metabolites poorly. The dominance of the Bacteroides MFG was again predicted by the model.

Data was also published for continuous culture of faecal samples with a pH shift added to the experiment. Figure 2 displays the model predictions compared to these experimental data. In this experiment, the population was more evenly distributed before the pH shift, after which the Bacteroides MFG dominated. The model captured this trend qualitatively, although quantitatively the predicted proportions did not match those observed experimentally.

The mean bias values for the model prediction of each measured SCFA using the Alpha parameter set demonstrated the high quality of model fit to these data (see Fig. 4 and Additional file 2: Table 1). However, using the Beta parameter set, acetate was overpredicted and butyrate underpredicted, without the dramatic change in the concentrations of these SCFAs that was observed experimentally after the pH shift.

A second pH shift experiment was performed with a faecal population from a different donor (Additional file 2: Fig. 2). The experimental and model dynamics were similar to those in Fig. 2, and the quality of model fit is displayed in Fig. 4 and Additional file 2: Table 1.

The next dataset used for comparison was that of Payne et al. [14]. These researchers used a three-compartment sequential fermenter, inoculated with faecal material from either an obese or a normal-weight child, to study the impact of high-energy, normal-energy and low-energy diets on the profile of the microbiota and the SCFAs produced.

For the first compartment in the experiment using the obese child’s faecal material (Fig. 3), total SCFA concentrations were predicted with good accuracy but low precision (see Fig. 4 and Additional file 2: Table 1). Acetate concentrations were underpredicted using both parameter sets, while butyrate was overpredicted. Propionate concentrations during continuous fermentation remained between 2 and 4 mM, while the model predicted continuously decreasing propionate concentrations throughout continuous culture. The predicted total SCFA concentration was similar to the observed data, although did not display the fluctuations observed experimentally within each dietary treatment. Propionate concentration was overpredicted for all media compositions in the latter two compartments.

Interestingly, the predictions for acetate and butyrate concentrations using the Beta parameter set appeared swapped compared to the experimental data: the model prediction for acetate concentration approximated the observed butyrate concentration, and vice versa. A possible explanation for this is metabolism of acetate and production of butyrate by the ButyrateProducers1 MFG, the abundance of which was consistently overpredicted by the model in all compartments when using the Beta parameter set (Additional file 2: Fig 3). For the Alpha parameter set, this relationship between acetate and butyrate predictions was not present throughout the experimental data, but could be seen from 20 h onwards in the latter two compartments (Fig. 3). Using the Alpha parameter set, the ButyrateProducers2 MFG was consistently overpredicted (Additional file 2: Fig 3), giving a similar possible explanation.

The SCFA and MFG results for the experiment with the normal-weight child’s faecal material were comparable to those of the first experiment (Additional file 2: Figs 4 and 5). Propionate concentration was predicted more accurately, but again an overprediction of butyrate concentration, with corresponding overprediction of butyrate-producing MFGs and underprediction of acetate, was seen in the latter two compartments.

The SCFA predictions of the model were consistent between the two parameter sets, with the exception of the acetate predictions. Use of the Beta parameter set resulted in acetate dynamics that shifted rapidly but minimally to new steady state values after a dietary shift (Additional file 2: Figs. 3 and 4). In contrast, use of the Alpha parameter set resulted in large and continued increases in acetate concentration over the course of the high-energy diet. This was likely due to the increased relative abundance of the Acetogen MFG seen in the use of the Alpha parameter set in all compartments under all diets, but particularly large under the high-energy diet (Additional file 2: Figs. 3 and 5). This increase was not seen using the Beta parameter set.

The MFGs showing relative abundance of > 10% were completely consistent between the simulations of the two experiments, demonstrating that diet rather than initial conditions was the major determinant of microbial profile in the model (Additional file 2: Figs. 3 and 5). However, this was not the case for the experimental data: the most abundant three MFGs determined from the observed data differed between the two experiments in eight of the nine measurements (low-energy conditions, compartment 1 was the only measurement that showed this consistency between the two experiments). The most notable failings of the model MFG predictions were the overprediction of the butyrate producing MFGs and the Acetogen MFG, and the inability to predict the high relative abundance of the NoButyStarchDeg and NoButyFibreDeg MFGs (which are capable of degrading resistant starch and NSP, with acetate rather than butyrate the SCFA produced).

No explicit data was available for the acetogen or the methanogen MFGs from these experiments; these were simply calculated as a proportion of the total Firmicutes and total bacteria, respectively (Additional file 1: Sect. 4). The SRB MFG was measured experimentally but showed a decrease from initial abundance in all cases but one: the third compartment on the low-energy diet, where modest increases of less than 1 log_10_ copies 16S rRNA gene g^−1^ were observed in both experiments. The model predicted a sustained decrease in the SRB MFG across all simulations, as the dilution rates of 6 d^−1^ in the first compartment and 3 d^−1^ in the latter two compartments were both greater than the maximum growth rate of the SRB MFG (2.78 d^−1^), preventing population growth.

The final dataset to which the model was compared was that of Belenguer et al. [15] (Additional file 2: Figs. 6–9). These authors also performed continuous cultures of faecal communities at pH 5.5 or pH 6 and recorded the SCFA production of the cultures. Unfortunately, insufficient data was recorded for the microbial community makeup to allow conversion of this data to microPop MFGs, so example initial MFG concentrations from Walker et al. [13] were used instead (Additional file 1: Sect. 4). While the example inocula used by the model were likely different to those in the experimental work, the model predictions for SCFA concentrations were mostly within the range observed experimentally. Interesting to note in these simulations was the pronounced differences between propionate and butyrate dynamics in the model predictions given different initial MFG concentrations. However, these differences were found only temporarily: when run for several hundred days, the model predictions for each of the three initial conditions used converged to identical values.

Neither parameter set outperformed the other on all the experimental data studied, either for metabolite concentrations or MFG abundance. An important caveat to the MFG profile comparisons in this section is that the quality of these comparisons is dependent on the assignment of experimental data to microPop MFGs, as described in Additional file 1: Sect. 4. The distribution of this data into MFGs is an approximation, based on similar work performed previously [11]. In simple terms, these comparisons represent the quality of fit of an eleven-member microbial community model to an incomplete measurement of a microbial population that was then assigned to these eleven groups. Far more assumptions were necessary in the establishment of this comparison than were necessary for the SCFA data, therefore the latter should be treated as the more reliable comparison between what was observed and what was simulated.

### Simulation of the colonic microbiota

**Fig. 5:**
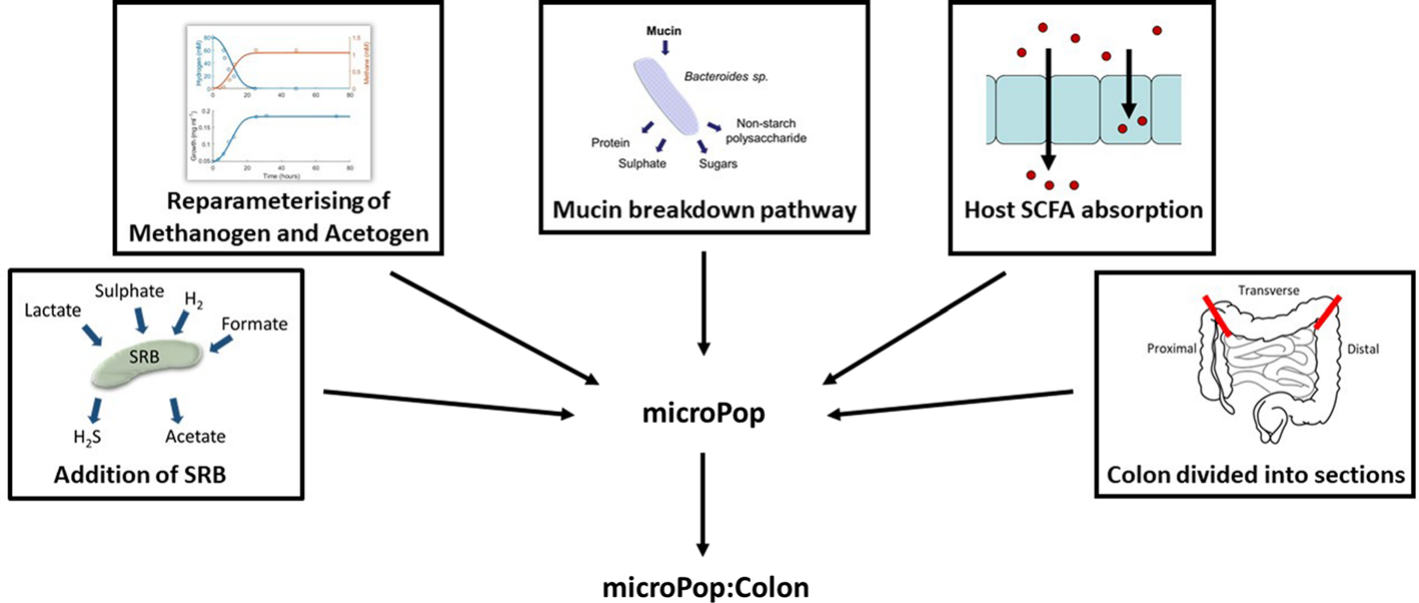
Diagrammatic explanation of the major adaptations made to microPop in the development of microPop:Colon. Note that division of the colon into sections was performed for the discrete version of the model only. See Methods and Additional file 1 for a detailed description

In order to study the colonic microbiota in silico, several adaptations were made to the original microPop structure, as detailed in the Methods and Additional file 1 and illustrated in Fig. 5. The adapted model, microPop:Colon, is able to simulate the colon in two different ways: either as a series of separate compartments, representing specific regions of the colon, each with their own individual luminal conditions; or, as a continuous model in which the passage of a fixed bolus of digesta is modelled over time and space simultaneously. In the former setup, the model is run to steady state (usually after a simulation period of at least 20 days) to provide predictions, whereas in the latter setup, the model simulates the colonic dynamics over the course of a single transit. The two structures are referred to as the discrete and continuous versions of microPop:Colon, respectively.

#### The discrete model

**Fig. 6:**
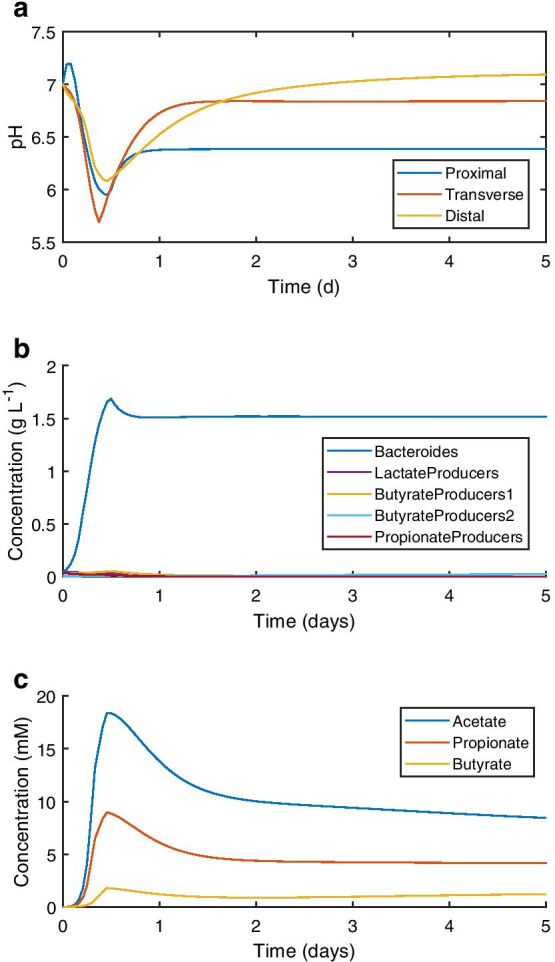
Selected results of the discrete version of microPop:Colon using the Alpha parameter set. **a** pH dynamics in each simulated colonic section over the first five days of simulation. **b** MFG dynamics of the five most abundant MFGs in the proximal section over the first five days of simulation. **c** SCFA concentrations in the distal section over the first five days of simulation

The discrete version of microPop:Colon simulates conditions in the proximal, transverse and distal colon. Selected dynamics from an example simulation are shown in Fig. 6, with steady state values obtained after 100 simulated days given in Table 1. Figure 6 displays the results using the Alpha parameter set.

**Table 1:** Comparison of simulation predictions from the discrete and continuous microPop:Colon models

	Discrete model (values at 100 hours)		Continuous model (values at set time points)		Literature data	References
	Alpha	Beta	Alpha	Beta		
pH	**6.4**	**6.4**	**5.7**	**5.7**	**6.37**, *6.61*, 7.04 (66 non-fasted subjects) **4.9/5.8**, *6.2/5.7*, 6.7/6* (2 recently deceased subjects) Mean 6.5, range 5-8 (20 fasted subjects)	[16] [18] [17]
	*6.8*	*6.8*	*5.8*	*5.7*		
	7.1	7.1	6.3	6.3		
SCFA concentrations (mM)						
Acetate concentration	**15.4**	**15.9**	**23.1**	**24**	97.5/98*^	[18]
	*11.7*	*12.9*	*26.7*	*33*	78.4/74.6*^	
	7.8	11	15.3	19.1	53.8/50.7*^	
Propionate concentration	**7.6**	**7.5**	**13.6**	**8.6**	34.5/30*^	[18]
	*5.7*	*5.7*	*15.2*	*11.4*	26.7/28.5*^	
	4.1	4.4	7.2	5.7	17.9/19.2*^	
Butyrate concentration	**0.3**	**0.2**	**5.6**	**7.7**	41.5/36*^	[18]
	*0.6*	*0.2*	*8.1*	*9.3*	35.8/32.1*^	
	1.3	0.4	3.9	4.2	16.4/25.8*^	
SCFA ratio (Acetate: Propionate: Butyrate)						
	**66:33:1**	**67:32:1**	**55:32:13**	**60:21:19**	56:20:24/60:18:22*	[18]
	*65:32:3*	*69:30:1*	*54:30:16*	*62:21:17*	56:19:25/55:21:24*	
	59:31:10	70:28:2	58:27:15	66:20:14	61:20:19/53:20:27*	
SCFA absorption (g d^-1^)						
Acetate	2.11	2.56	4.54~	5.45~	95% (≃9.4 g d^-1^ total for Alpha discrete and ≃13 g d^-1^ total for Beta discrete; ≃16.3 g d^-1^ total for Alpha continuous and ≃17.3 g d^-1^ total for Beta discrete) of total produced SCFAs	[19]
Propionate	1.63	1.66	3.64~	2.78~		
Butyrate	0.43	0.15	2.81~	3.27~		
Total	4.17	4.37	10.99	11.5		
MFG relative abundance (%)^#^						
Bacteroides	**98**	**97**	**48**	**27**	23 (mean value; Bacteroidetes) 11 (mean value; Bacteroides) 16, 35 (mean values using different techniques; Bacteroidetes) 26, 3 (mean values; lean, obese individuals; Bacteroidetes)	[67] [68] [69] [70]
	*95*	*95*	*42*	*24*		
	88	89	42	25		
NoButyStarchDeg	**<1e−10**	**<1e−10**	**0.2**	**0.2**	4 (mean value; *Ruminococcus bromii*)	[71]
	*<1e−10*	*<1e−10*	*0.1*	*0.2*		
	<1e−10	1e−10	0.1	0.2		
NoButyFibreDeg	**<1e−10**	**<1e−10**	**0.1**	**0.2**	11, 27 (mean values; lean, obese individuals; *Ruminococcus*)	[70]
	*<1e−10*	*<1e−10*	*0.2*	*0.2*		
	<1e−10	<1e−10	0.2	0.2		
LactateProducers	**<1e−10**	**1.8**	**20**	**36**	5 (mean value; Actinobacteria) 4 (mean value; Actinobacteria) 2, 5 (mean values using different techniques; Bifidobacteria) <1, 4 (mean values; lean, obese individuals; Actinobacteria)	[67] [68] [69] [70]
	*<1e−10*	*3.5*	*21*	*39*		
	<1e−10	7	21	39		
ButyrateProducers1	**<1e−10**	**0.9**	**16**	**22**	3 (mean value; *Roseburia*) 4 (mean values; *Roseburia intestinalis*)	[67] [72]
	*<1e−10*	*2*	*19*	*23*		
	<1e−10	4	19	23		
ButyrateProducers2	**2**	**<1e -10**	**2**	**1**	5 (mean value; *Faecalibacterium*) 2 (mean value; *Faecalibacterium prausnitzii*)	[67] [72]
	*4*	*<1e−10*	*2*	*1*		
	11	<1e−10	3	1		
PropionateProducers	**<1e−10**	**<1e−10**	**14**	**13**	4, 4 (mean values using different techniques; Veillonella)	[69]
	*0.2*	*0.2*	*15*	*11*		
	0.5	0.7	15	11		
ButyrateProducers3	**<1e−10**	**<1e−10**	**0.1**	**0.1**	0.04 (mean value; *Eubacterium hallii*) 0.5 (mean value; *Eubacterium hallii*)	[73] [72]
	*0.001*	*0.003*	*0.1*	*0.09*		
	0.004	0.004	0.1	0.08		
Acetogen	**<1e−10**	**<1e−10**	**0.04**	**0.04**	Found in all regions of the colon, but abundance varies slightly between regions and individuals (10^4^-10^5^ gene copies g^-1^ in mucosal biopsies). More abundant than methanogens and SRB in faeces. 1 (mean value; *Blautia*)	[44] [74] [67]
	*<1e−10*	*<1e−10*	*0.05*	*0.03*		
	<1e−10	<1e−10	0.05	0.03		
Methanogen	**<1e−10**	**<1e−10**	**0.05**	**0.06**	Found in all individuals, but not found in all regions of each individual. Increased population size distally.	[44]
	*<1e−10*	*<1e−10*	*0.03*	*0.06*		
	0.06	0.01	0.04	0.2		
SRB	**<1e−10**	**<1e−10**	**0.05**	**0.05**	Found in all regions of the colon, but abundance varies widely between regions and individuals (10^2^-10^9^ gene copies g^−1^ in mucosal biopsies) 0.02 (mean value; *Desulfovibrio*) 0.1, 0.03 (mean values; lean, obese individuals; *Desulfovibrio*)	[44] [75] [70]
	*<1e−10*	*<1e−10*	*0.04*	*0.04*		
	<1e−10	<1e−10	0.06	0.05		

Font style denotes values specific to the **proximal**, *transverse* and distal regions of the colon

^*^Data from Macfarlane et al. [18] is displayed as Subject1/Subject 2, for the two subjects analysed

^mM concentrations were calculated from mmol kg^−1^ reported data assuming 1 kg faecal contents has approximately 1 L volume [76]

^#^Literature data for relative abundance was obtained from studies on faecal samples using a wide variety of techniques and various groups of faecal donors. Unless otherwise stated, the data is for healthy adults and the mean value for the indicated taxonomic group has been included, which corresponds to the assigning of MFGs to taxa in Kettle et al. [11]. These values are included for comparison only and should not be interpreted as representative means for all individuals

~ Values from the model were tripled to give the daily total, since only one third of the daily metabolite influx into the colon was modelled using the continuous model

Figure 6a displays the changes in pH over the course of the first five days after model initiation. The model was initiated with microbial community data from Walker et al. [13] (Additional file 1: Sect. 4), initially present in the proximal compartment only. Due to the high initial availability of substrates (Additional file 1: Sect. 3), there was a phase of rapid population growth and metabolism during the first simulated day. The resulting production of SCFAs caused a rapid decrease in pH, most notable in the proximal and transverse compartments where the microbial population was at greatest abundance during this time. Due to a depletion of substrate, host buffering, SCFA absorption and washout, the pH climbed gradually thereafter, approaching steady state values between pH 6.3 and 7.2, as observed in vivo [16, 17].

Figure 6b shows the concentration in the proximal compartment of the five most abundant MFGs over the first five days after model initiation. The abundances of the remaining MFGs were too low to be distinguished in this plot. The Bacteroides MFG dominated this compartment from model initiation onwards, including at steady state (Table 1).

As is clear from the pH dynamics shown in Fig. 6a, there was a drop in pH in all three compartments during the first day of simulation, due to a rapid increase in SCFA concentration. Figure 6c shows the change in SCFA concentration in the distal compartment over the first five days of simulation. Increases here were the result of SCFA inflow from the transverse compartment, alongside SCFA production from what substrates were still available. SCFA removal was due to host absorption and washout.

Under the conditions used for this simulation, the acetogen and SRB MFGs were washed out after small population increases over the first two days of the simulation. This was likely due to the dilution rates used for the simulation, which were high relative to the maximum growth rates of these MFGs, and competition for organic substrates between the acetogen MFG and other saccharolytic MFGs. The methanogen MFG was washed out of the proximal and transverse compartments, likely due to the lower pH, with no methanogen growth possible below pH 6 and limited growth below pH 6.9. However, the methanogen MFG achieved steady state concentrations in the order of 0.001 g L^−1^ in the distal compartment (Table 1).

The pH predictions of the model for each compartment were within 0.2 pH units of literature measurements (Table 1). All SCFA concentration predictions of the model were lower than those measured in sudden death victims (Table 1; [18]). Perhaps more important is the ratio of SCFAs, often stated as approximately 60:20:20 for acetate:propionate:butyrate in vivo [19]. Measurements from sudden death victims were comparable to this ratio (Table 1). The discrete model predicted a mean ratio across the three compartments of 63:32:5 using the Alpha parameter set, implying that acetate and propionate were overpredicted at the expense of butyrate. Absorption of SCFAs by the host was underpredicted by the model. The absorption parameters were calculated from perfusion studies rather than observational research; thus, the rate of absorption may be different under normal colonic conditions. Moreover, the rate of absorption in the model increases linearly with the colonic volume, which was assumed fixed for these simulations. An increased colonic volume induced by digesta influx would increase the rate of absorption in the model.

The model predicted at least 88% relative abundance of the Bacteroides MFG in all three compartments using both parameter sets (Table 1). While much evidence in the literature also predicts that this MFG should be highly abundant, 88% is significantly higher than the 3–35% estimates available in the literature and was a failing of the discrete model (see Table 1 for literature references). The dominance of the Bacteroides MFG also resulted in minimal relative abundances of MFGs that compete for substrates with the Bacteroides MFG, which includes all but the methanogen and SRB MFGs. As such, the estimates of MFG relative abundance did not compare well with measurements in the literature (Table 1).

The predictions of the discrete model using the two parameter sets were similar in most respects. The most notable discrepancy between the two sets was in the SCFA ratio, with the model predicting higher ratios of acetate when using the Beta parameter set. Regarding MFG relative abundances, use of the Beta parameter set resulted in higher predictions for the LactateProducers and ButyrateProducers1 MFGs, whereas use of the Alpha parameter set resulted in higher predictions for the ButyrateProducers2 MFG.

Although the pH profile predicted by the model was representative of in vivo data, the low SCFA concentrations, low SCFA absorption and the dominance of all colonic sections by the Bacteroides MFG mean that substantial changes would be required in order to reflect the colonic environment. One major assumption made in the use of the discrete model was that the steady state values would give the best prediction of in vivo conditions. The entry of digesta into the colon is not continuous and expulsion of faeces is periodic, in contrast to the model setup. Therefore, it is not feasible to expect that the variables predicted by the model at steady state will remain at this state in vivo. To challenge the steady state assumption, a continuous version of the model was developed.

#### The continuous model

**Fig. 7:**
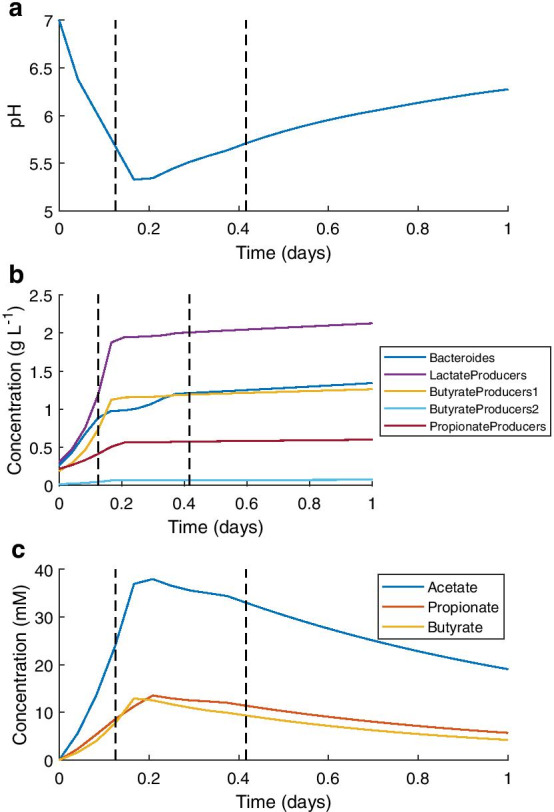
Selected results of the continuous version of microPop:Colon using the Beta parameter set. The vertical dashed lines indicate timepoints corresponding to the values in Table 1. **a** pH dynamics over the single day simulation. **b** MFG dynamics of the five most abundant over the single day simulation. **c** SCFA concentrations over the single day simulation

To match the discrete model, a colonic transit time of one day was chosen for the continuous model. This was convenient as it matched the model time unit of days, and was reflective of in vivo transit times [17, 20]. Digesta was assumed to progress continuously along the colon over time. The dilution rates calculated from volumes used for the discrete model allowed calculation of mean residence times in each colonic region in the model: approximately 14% (3.4 h) for the proximal colon, 32% (7.7 h) for the transverse colon and 54% (12.9 h) for the distal colon. Therefore, the model estimates for each region were taken at 3, 10 and 24 h. The concentration of inflowing substrates was set at one third of the daily influx for the discrete model, assuming this represents one of three daily meals. The continuous secretion of mucins and bicarbonate by the host was also set at one third of their daily value. The initial microbial population was again taken from Walker et al. [13], as for the discrete model. The estimates of the model at the regional time points are given in Table 1. Figure 7 displays the predictions of the model using the Beta parameter set.

The pH profile followed the expected pattern established by the discrete model: there was a rapid initial decrease in pH caused by microbial production of SCFAs, which was followed by a more gradual return towards neutral pH caused by SCFA absorption and bicarbonate secretion (Fig. 7a). However, the pH did not return to neutrality during the 24-h simulation, finishing at pH 6.3 using both parameter sets. This is lower than the distal colonic pH estimates found in the literature (Table 1), perhaps reflecting inaccuracies in the bicarbonate buffering aspect of the continuous model.

As shown in Fig. 7b, the five MFGs showing the greatest population growth were identical to those shown in Fig. 6b for the discrete model. The remaining MFGs attained population sizes too small to be distinguished in this plot. There was a more even distribution of MFGs in the continuous model predictions using either parameter set than was seen using the discrete version of the model. The MFG relative abundance values from the continuous model were similar when using the Alpha or Beta parameter sets, with the exception of the Bacteroides and LactateProducers MFGs. The Bacteroides MFG attained 42–48% relative abundance throughout transit when using the Alpha parameter set compared to the LactateProducers 20–21%, whereas these positions were reversed when using the Beta parameter set, with Bacteroides relative abundances of 24–27% and LactateProducers relative abundances of 36–39% (Table 1).

The continuous model predictions for SCFA concentrations were similar to the steady state predictions of the discrete model, although higher maximum concentrations were achieved by the continuous model (Fig. 7c). Use of the Beta parameter set resulted in higher acetate concentrations than did the Alpha parameter set, and an SCFA ratio more similar to that observed in vivo (Table 1). Once again, the SCFA concentrations were lower than the literature estimates for these values.

Absorption of SCFAs was greater using the continuous model than the discrete model, with two thirds of the available SCFAs being removed from the colon by the host during transit. However, this was still less than the 95% absorption rate stated in the literature [19].

Overall, each of the four model runs analysed here (the discrete and continuous models with each parameter set) gave the best predictions of the four for some aspects of the comparison to literature values in Table 1. However, the superiority of the discrete model was only seen for predictions of pH. The SCFA concentration and absorption predictions were poor, and the domination of all colonic sections by the Bacteroides MFG meant that little could be drawn from the microbial side of the discrete model. Contrastingly, the continuous version of the model, despite predicting lower pH values, was more accurate in predicting SCFA concentrations, absorption and the relative abundance of MFGs.

The consistent underprediction of SCFAs by each of the models could be remedied by a greater concentration of protein, NSP and resistant starch entering the colon. However, none of the model runs were able to achieve predictions for the NoButyStarchDeg and NoButyFibreDeg MFGs within an order of magnitude of the observed relative abundances, making this the only consistent failing of the model that could not be explained by insufficient substrate availability. Since these MFGs were also underpredicted in the comparisons to experimental data in the "Comparison of model predictions to experimental data" section, the accuracy of the parameter values for these MFGs likely requires further investigation.

#### Investigation of varied colonic sulphate availability

The microPop:Colon model was developed for use as a tool to quickly provide predictions on the effect of changes in substrate availability on colonic dynamics. As our interest was in the hydrogenotroph dynamics in the colon, it was considered what the role of inflowing sulphate quantity was on the SRB population and SRB hydrogen sulphide (H_2_S) production in the colon. Previous research has shown both positive and neutral results of increased dietary sulphate increasing the colonic SRB population [21–25]. The hypothesis was that changes in sulphate inflow would have a negligible influence on SRB population size and H_2_S production in the model. The reasoning was that the sulphate released during mucin metabolism would be in excess of what can be metabolised by the small SRB MFG population in the colon, therefore sulphate would not be limiting, and additional sulphate would have no effect. To investigate this, microPop:Colon was run with varied sulphate inflow concentrations and transit times.

The discrete and continuous versions of the microPop:Colon model were run as described in the previous sections, using the Alpha parameter set and with sulphate inflow the only substrate that was varied. Conditions compared were as follows: zero sulphate (NoS), where sulphate may be derived from cross-feeding on the breakdown products of mucin only; low sulphate availability (NormS), for which the results of the previously performed model runs with 0.86 g L^−1^ d^−1^ sulphate available were used; and high sulphate availability (HighS), where the sulphate availability was increased by a factor of 10 from the NormS case. To investigate the influence of transit time on the results, colonic transit times of one day (as investigated previously), two days and four days were simulated.

Using the discrete model, variations in sulphate resulted in a maximum concentration change of less than 0.02% for those MFGs that avoided washout, including the hydrogenotrophs. Steady state differences of less than 10^–4^ g L^−1^ were observed for all metabolite concentrations, with the exception of sulphate, which increased with increasing influx levels.

The results of the continuous model showed greater differentiation between sulphate and transit time conditions (Table 2). Changes in the non-hydrogenotrophic MFGs and the acetogen MFG between sulphate influx levels were minimal, as were changes in SCFA concentrations. The concentration of the methanogen MFG increased under the NoS influx level and decreased under the HighS influx level, the magnitude of this change increasing with increasing transit times. Conversely, the concentration of the SRB MFG decreased by at least 19% under the NoS influx level and increased by 1–2% under the HighS influx level, with H_2_S concentration changes following the SRB trend.

**Table 2:** Summary of changes in microbiota and metabolite concentrations with varied sulphate influx and transit times. Percentage changes to one significant figure compared to the results under NormS conditions are shown, after the full transit time

Colonic transit time	1 day		2 days		4 days	
Sulphate influx	NoS	HighS	NoS	HighS	NoS	HighS
Non-hydrogenotrophic MFGs	< 0.02	< 1e−3	< 0.02	< 2e−04	< 0.01	< 3e−04
Acetate	− 5e−03	+ 1e−04	−4e−03	+ 1e−06	−3e−03	+ 7e−05
Propionate	− 2e−03	+ 3e−05	− 4e−04	+ 1e−05	+ 8e−04	− 1e−04
Butyrate	− 6e−03	+ 1e−04	− 3e−03	+ 5e−05	+ 2e−04	− 9e−06
Acetogen MFG	+ 0.04	− 1e−3	+ 0.04	− 8e−04	+ 0.01	+ 2e−04
Methanogen MFG	+ 0.7	− 0.03	+ 3	− 0.2	+ 10	− 0.7
SRB MFG	− 19	+ 1	− 25	+ 2	− 25	+ 2
H_2_S	− 39	+ 2	− 35	+ 3	− 28	+ 2

Thus, the discrete and continuous models were not consistent in their conclusions on the effect of varied sulphate influx on the SRB MFG. The discrete model predicted washout of the SRB MFG under all conditions simulated, whereas the continuous model predicted that increased sulphate would result in incremental increases in SRB and H_2_S concentrations, while removal of sulphate inflow would result in substantial decreases in both these quantities.

## Discussion

The modelling work described here builds on the work of Kettle et al. [11], developing and applying the microbial community model microPop [10] for the study of the human colonic microbiota. Several features were added to the model, encompassing both human and microbial metabolites and functions. The increased attention given to hydrogen cross-feeders in this work increases the model’s potential to provide useful predictions in the analysis of these microbes.

The number of in vitro data sources used for model validation was limited by the requirement for detailed information on study design and time-course measurements of metabolite and microbial concentrations. However, the three sources and 14 independent experiments used allowed for comparison of the model predictions to microbial data for the majority of MFGs in the model, and against concentration data for five microbial metabolites, namely acetate, butyrate, propionate, lactate and formate.

The accuracy of the model predictions varied between parameter sets. Overall, no clearly superior parameter set was established from the validation runs: while the Beta parameter set of Wang et al. [12] appeared superior to the original Alpha parameter set of Kettle et al. [10] in most cases, its poor performance in the pH shift experiments of Walker et al. [13] prevents its recommendation as the more accurate parameter set for all cases. A similar conclusion was reached in the microPop:Colon simulations: predictions of the model using either parameter set were similar, with similar flaws. In the future, it may be possible to derive a new parameter set, incorporating the latest knowledge of the MFGs, which shows better performance than either current parameter set. Alternatively, it could be possible to select the parameter set based on knowledge of the specific strains in the inoculum to be simulated.

The viable and optimal pH range of each MFG was altered between the Alpha and Beta parameter sets. As pH varies between 5 and 7.5 in the simulations of the in vitro experiments and for the microPop:Colon runs, this change has a strong effect on growth. Moreover, substantial alterations were made to the maximum growth rate of the Bacteroides MFG on protein, NSP and resistant starch. Due to the dominance of this MFG in many of the simulations detailed here, the differing model predictions between parameter sets were to be expected.

The original setting of parameter values in the Alpha set by Kettle et al. [11] and of the more recent Beta set was performed either from monoculture experimentation or assumptions based on the literature. As a result of this use of monoculture parameters, the model inherently assumes that monoculture metabolic parameter values are also accurate in a co-culture environment. Experimental and modelling evidence in the literature both supports and opposes this assumption, with monoculture parameters sufficient for certain applications to co-culture [26, 27], but with co-culture fitted parameters necessary for more complex communities [28, 29]. Unfortunately, the derivation of parameter values for co-culture growth is challenged by the difficulty of successfully co-culturing multiple strains in vitro and extracting the contributions of each to net metabolite flux. The current model version is constrained by this lack of data.

There are obvious limitations in assuming that a single set of parameter values is representative of an entire functional group: clearly more variation exists between strains in the same MFG than is captured using this assumption. Kettle et al. [11] addressed the need for greater diversity of metabolic capabilities by running their model with multiple strains in each MFG, each of which had stochastically varied parameter values within a certain range. Stochastic variation within each MFG was not performed here, but would be a natural next step in the analysis of the model. While this would likely result in quantitative changes in the relative abundance of many MFGs, we speculate that it would be unlikely to qualitatively change aspects such as the washout of hydrogenotrophs in the discrete model, or the overall dominance of the Bacteroides MFG seen in most of the simulations.

The major addition to the original microPop model described here was the inclusion of the SRB MFG and the alterations to the modelling of the other hydrogenotrophic MFGs. A full comparison of the developed model with the original microPop model has not been presented here, mainly due to a lack of data around hydrogenotroph dynamics: none of the data sources used here measured all three hydrogenotrophic MFGs and their associated metabolites. The novel SRB MFG was washed out in all simulations in the "Comparison of model predictions to experimental data" section, due either to an absence of sulphate or mucin (Walker et al. [13] and Belenguer et al. [15] datasets) or high dilution rates (Payne et al. [30] dataset), thus this MFG had a negligible effect on the model predictions. The changes made to the other two MFGs also had little effect due to the incremental nature of the changes and the low abundance of these MFGs. For example, when the predictions for the developed model shown in Figs. 1 and 2 were compared to those of the original microPop version, the results were nearly identical, but for the acetate and acetogen MFG predictions. The developed model predicted lower acetate concentrations in both simulations (mean bias of developed model: 10.6 mM and 0.7 mM for Figs. 1 and 2, respectively; corresponding mean bias of the original model: 11.1 mM and 1.1 mM, respectively), likely due to a lower abundance of the acetogen MFG in the predictions of the developed model.

Another challenge faced by the creators of the original microPop model was in determining the initial abundance of MFGs. Kettle et al. [11] determined the abundance of the acetogen MFG in faecal samples as a proportion of counts from two Firmicutes-targeting 16S rRNA probes, and the abundance of the methanogen MFG simply as a proportion of the total bacterial counts. While some of the probes used matched the corresponding MFGs well (e.g. Bac303 probe for the Bacteroides MFG), others (e.g. acetogen, methanogen, NoButyStarchDeg and NoButyFibreDeg MFGs) were not as well targeted and must be considered ‘best guesses’ [11]. The fact that the simulated abundances of NoButyStarchDeg and NoButyFibreDeg MFGs frequently did not match the observed abundances in the experimental data may be a result of poor approximation between microbial data and MFGs. The use of functional gene counts, suggested by Kettle et al. [11], would improve the accuracy of these approximations.

The development of microPop:Colon required the inclusion of environmental factors and their influence on MFGs. The inclusion of host SCFA absorption allowed the effect of the colonic microbiota on the host to be quantified. The proportion of total SCFAs absorbed was consistently lower than observed in vivo, which could be due to several factors. Firstly, the absorption parameter used in microPop:Colon was calculated using results from perfusion experiments; colonic absorption under normal conditions may take place at a different rate. Secondly, the absorption rate in microPop:Colon was dependent on the volume of the colonic section (for the discrete model) or of the modelled digesta (for the continuous version), with greater volumes resulting in greater SCFA absorption. Estimates of adult colonic volume in the literature vary widely, from the 3.02 L used here [31, 32] to a fasting volume of as little as 0.5 L [33], thus the volume estimate may be inaccurate and biasing the absorption rate.

microPop:Colon also considered bicarbonate secretion by the host, and subsequent buffering of the colonic environment. This is one of the strengths of the model, as it allows for a more physiological representation of pH than would be possible if pH were fixed. However, it was challenging to find consistent estimates of colonic bicarbonate secretion in the literature. Bicarbonate ions are exchanged for SCFAs at the colonic epithelium, but also for other ions such as chloride, which were not modelled [34, 35]. The constant influx of bicarbonate used in the model was based on measurements from perfusion experiments, which may not be representative of normal colonic function.

Although the inclusion of pH in the model adds functionality, there is also potential for this to contribute to errors in the model predictions. If the pH value is incorrect, then this will influence the growth and metabolism of the modelled MFGs, resulting in variation in the production of pH influencing metabolites. This feedback mechanism should be considered in interpreting model results and should also be an area for future development and testing of the model.

Another aspect of the model that defies consistent estimation is transit time. For microPop:Colon, a mean transit time of 24 h was chosen based on measurements in the literature [16, 20]. However, other research on healthy individuals has found median colonic transit times as high as 72 h, with individual transit times ranging from 14 to 132 h [36–38]. These transit times were also not consistent between subject groups divided by gender or by age [20, 36].

Transit time has been shown as a determining factor in hydrogenotroph abundance [25, 39]. Both referenced studies saw a negative correlation between methanogen abundance and SRB abundance, with the former seen at higher abundances with slower colonic transit. The abundance of Archaea has been associated with harder stools, indicative of longer transit times [40, 41]. There is also evidence that the presence of colonic methane can slow colonic transit [42]. The washout status of the acetogen and SRB MFGs were unaffected by varied transit time in the discrete model, but the methanogen MFG achieved increased steady state concentrations in the transverse and distal compartments at the higher transit times simulated in Sect. 3.2.3.

One important omission of the model was the mucous layer. Previous models for colonic microbiota dynamics, such as that of Muñoz-Tamayo et al. [31], have included compartments representing the mucous layer and shown different dynamics in this habitat. The mucous layer allows certain microbes to adhere to mucosal structures, thus increasing their residence time in the colon and generating a population distinct from that of the lumen [43]. The lower dilution rates associated with the mucous layer could be important in the persistence and greater abundance of the slower growing MFGs, such as the hydrogenotrophs [44]. As the mucous layer was absent from microPop:Colon, it should be considered a model for the luminal dynamics in the colon, rather than the entire colonic environment.

microPop:Colon predicted no increase in the SRB MFG concentration in the discrete model and only minimal increases in the continuous model with increased sulphate influx. This, alongside the inverse relationship between the SRB and methanogen MFGs in terms of population size in this investigation, suggests that competition between these MFGs for hydrogen or formate may be present. This relationship was not seen with the acetogen MFG, perhaps due to its additional ability to metabolise carbohydrates. Competition between the hydrogenotrophic MFGs has been postulated previously but no consensus has yet been reached on the extent to which this occurs in the colon [4, 5].

The microbial community in microPop:Colon was shown to vary from the profile seen in vivo (Table 1). However, substantial differences were seen between parameter sets and between the discrete and continuous models, despite identical initial abundances of each MFG. The specific profile of the microbiota, including hydrogenotrophs, has been repeatedly shown to vary between individuals and within individuals over time [44–47], so it is unsurprising that models for this complex population exhibit high variability. This variability could potentially be minimised given a reliable estimate of the initial MFG concentrations. Currently, microPop:Colon uses faecal abundance data as a proxy for the proximal colon, due to a lack of data on the proximal colonic population. However, the population at the beginning of the colon is known to differ from the faecal population [48]. Therefore, it is not expected that the faecal abundance data used to initiate microPop:Colon is representative of the proximal colonic microbiota. However, this estimate has been used in the absence of more appropriate data, and the initialisation of microPop:Colon with an initial microbial population much lower than the carrying capacity allows for the establishment of a population with a profile appropriate to the local environment.

In the future, it may be possible to use faecal abundance data to give an estimate of the proximal colonic population using microPop:Colon. Inverting the model, so that data such as faecal pH, MFG and metabolite concentrations are the input, could allow a reverse simulation or Markov Chain Monte Carlo estimation to be conducted in which the proximal colonic population could be predicted, to within some degree of accuracy. Such a model would require rigorous validation of the current model against human data, as the prediction quality of the reverse model would be dependent on its accuracy in its current form.

There are several existing models for microbial and metabolite dynamics in the colon. Table 3 gives an overview of selected mathematical models that perform similar roles to microPop:Colon. The novel aspects of microPop:Colon are the option to run the model either in discrete, compartmentalised form, or with the continuous version. The inclusion of SRB and the focus on hydrogenotrophs are also new additions to the field, and the diversity of the microPop MFGs sets it above other models in terms of applicability to microbial abundance data. Areas where the model is less sophisticated than previous publications are its lack of a mucous layer and a gaseous phase, as well as a less comprehensive catalogue of metabolites than is possible using genome-based metabolomic reconstructions (e.g. [49]). Moreover, water is absorbed from the lumen as digesta passes along the colon [50], which results in changes in metabolite concentration, digesta viscosity and volume. As water absorption was not included in microPop:Colon, the effects of this dynamic on microbial and metabolite concentrations were not captured. Other models for the colon have included water absorption [51], and this would be a good addition to future versions of microPop:Colon.

**Table 3:** Summary of selected mathematical models for the colonic environment

Publication (model name)	No. of MFGs	No. of metabolites	Key features
Muñoz-Tamayo et al. [31]	4	13	Lumen, mucous and gaseous compartments over three colonic sections
Motelica-Wagenaar et al. [2]	10	24	Proximal colon only, adaptation of Muñoz-Tamayo et al. [31] Parameterised from in vitro data Impact of transit time, substrates, pH, and presence of methanogens investigated
Moorthy et al. [52]	4 (multiple strains within MFG)	10	One-dimensional spatially continuous adaptation of Muñoz-Tamayo et al. [31] Only carbohydrate substrates considered No gaseous phase Varied transit times, fibre intakes and number of strains in the primary degrader MFG investigated
Bauer et al. [3] (BacArena)	7	50 +	Two-dimensional cellular automaton constraint-based reconstruction and analysis (COBRA) model Includes cellular motility and mucin layer
Van Hoek and Merks [49]	Single supra-organism used	50 +	Two-dimensional cellular automaton COBRA model Evolutionary component of metabolic pathway gain/loss No specific MFGs, theoretical organisms with widely varied metabolic capabilities Varied transit times investigated
Cremer et al. [51]	2	7	One-dimensional mechanistic model Includes relationship between SCFA production, pH and microbial growth Models water absorption and peristaltic movement of the colon wall
Labarthe et al. [53]	4	12	A unification of Muñoz-Tamayo et al. [31] with Cremer et al. [51], also including microbial active motion Diet, mucous, chemotaxis and peristalsis variations investigated
microPop:Colon	11	20	Spatially discrete or continuous versions Responsive pH Host secretion and absorption Inclusion of mucins, but no mucous layer or gaseous phase Inclusion of SRB

## Conclusions

Adaptations to the original microPop model of Kettle et al. [10] have been presented that allow it to be applied to the human colon while retaining the qualities of the original model. The inclusion of all three hydrogenotrophic MFGs is a strength of the model, as is the option to use either the discrete or continuous versions. The model predictions compared well to literature data in some respects, while pointing to areas for model improvements in others. Perhaps the most pressing future inclusion would be the addition of a mucous layer compartment to microPop:Colon, to allow the important role of this habitat to be captured. The potential for the model to address biological questions in its current form, such as the role of sulphate in the colon, has been demonstrated.

microPop:Colon represents a tool for the further interrogation of experimental data or investigation of hypotheses on the behaviour and function of the human colonic microbiota. Once validated against experimental data for a specific in vitro system, the model could be used to analyse the data from a different perspective. With further validation against in vivo data, the model could be used to make predictions about the effects of dietary interventions, probiotic performance, or antibiotic resistance on the microbiome. Some other example uses of the model include: deriving an estimate for the amount of acetate produced via reductive acetogenesis during a faecal fermentation study; estimating the effect of increased dietary resistant starch on the colonic microbiota and SCFA absorption; or, providing a prediction for the best candidate prebiotics to elicit a butyrogenic effect before beginning more expensive in vitro tests.

The advantages of microPop:Colon over its in vitro and modelling alternatives are its rapid results, low cost and the ease with which new functionality can be built in. Disadvantages include the limited depth to which microbes and metabolites can currently be interrogated, in comparison to metagenomics and metabolomics approaches, and the degree of abstraction necessary, leaving the quality of model predictions dependent on the validity of its simplifying assumptions. Thus, the model should be viewed as complementary to experimental work. There is clear potential for both microPop and the colonic expansion of the model to be grown, with inclusion of further metabolites and MFGs and further comparison to in vitro and in vivo data. The model has the potential to benefit microbiome research both by guiding experimental research through the provision of experimentally testable hypotheses, and in application to questions that cannot feasibly be addressed experimentally.

## Methods

### Consideration of hydrogen cross-feeding in microPop

The microbial community modelling tool microPop [10] models microbial activity and metabolite concentrations by accounting for the growth and metabolism of several MFGs, each of which is representative of a subset of the wider microbial community. For each of these MFGs, microPop implements information on the different metabolic pathways available to the MFG and the corresponding parameter values for these pathways, including maximum growth rate, half-saturation constants, yield factors and stoichiometries. microPop also considers the pH preferences of each MFG, scaling metabolic activity according to environmental pH. microPop is based on Monod kinetics, constructing and solving a system of ordinary differential equations for given initial conditions relating to each MFG and each metabolite. Full details of the equations used are included in Additional file 1: Sect. 1. The MFG kinetic parameter values used here were based on two different parameter sets: the Alpha set, utilising the original values of Kettle et al. [10]; and a Beta set, based on newly published values [12]. These values are included in Additional file 1: Sect. 7. Use of both parameter sets allowed for comparison of the predictions of the two parameterisations.

The main alterations to the microPop MFGs performed here involved the representation of hydrogenotrophs. microPop included hydrogenotrophic pathways in a methanogen and an acetogen MFG. Changes to the original methanogen MFG parameter values were made as detailed in Additional file 1: Sect. 2. Alterations were also made to the hydrogenotrophic pathway of the acetogen MFG: as previous research has demonstrated a hydrogen uptake threshold for these bacteria [54], the threshold model for *Blautia hydrogenotrophica* of Smith et al. [55] was used instead of the original microPop hydrogenotrophic pathway for the acetogen MFG. Finally, a novel SRB MFG was added to the model. This MFG included a hydrogenotrophic pathway and a lactate metabolism pathway, both based on the *Desulfovibrio vulgaris* model of Smith et al. [56], as well as formate metabolism. Full details of the three hydrogenotrophic MFGs can be found in Additional file 1: Sect. 2.

### Comparison of microPop to experimental data

A predecessor model to microPop has previously been validated against experimental data from faecal fermentations [11, 13]. In order to validate microPop after the alterations to the hydrogenotrophic MFGs, model predictions were compared to continuous faecal culture data from three independent sources. Data was sampled from Walker et al. [13] (the same data used in previous model validations [11]), Belenguer et al. [15] and Payne et al. [14] using image capturing and graphical input software in MATLAB (The MathWorks; www.mathworks.com). SCFA and MFG measurements were converted into microPop units from information provided in the publications (Additional file 1: Sects. 3 and 4).

### Adaptation of microPop to the human colonic environment

To perform the theoretical study of hydrogen cross-feeding and model the activity of the microbiota in the human colon, several further alterations to microPop were introduced. The colon version of the model is referred to as microPop:Colon.

#### Physiology

In the discrete model, the colon was divided into three sequential compartments, representative of the proximal, transverse and distal sections. To make the model reflective of the colonic environment, an overall dilution rate of 1 d^−1^ was chosen for the entire colon, reflective of mean transit times in the literature [17, 20]. The dilution rate was scaled in each compartment according to the relative volume of the compartment. A fixed colonic volume of 3.02 L was assumed, with 0.41, 0.98 and 1.63 L volumes in the proximal, transverse and distal colons, respectively, based on previous calculations [31]. Although the volume of the colon will vary in vivo, these assumptions on the volumes only affected compartmental transit time, metabolite absorption and bicarbonate secretion in the model.

#### pH

pH variation based on the metabolism of the microbiota and host activity was identified as an important inclusion. Thus, the charge balance model structures of Batstone et al. [57] and the simplified version of Muñoz-Tamayo et al. [58] were adapted for the colonic environment. Full details of the pH calculations are given in Additional file 1: Sect. 6. Briefly, it was assumed that a charge balance is maintained between positively charged ions (H^+^ ions and miscellaneous other cations) and negatively charged ions (dissociated SCFAs, bicarbonate and hydroxide). Moreover, it was assumed that the host buffers the colonic lumen via secretion of bicarbonate ions, absorption of SCFAs and secretion and absorption of CO_2_. Absorption of SCFAs is described in Sect. 2.3.3. Bicarbonate and CO_2_ was modelled to adhere to the equilibrium equation:

$$K_{{a,CO_{2} }} s_{CO_{2}} - s_{{HCO_{3}^{ - } }} s_{{H^{ + } }} = 0,$$

where $K_{{a,CO_{2} }}$ is the equilibrium constant for CO_2_, $s_{CO_{2}}$, $s_{{HCO_{3}^{ - } }}$ and $s_{{H^{ + } }}$ are the concentrations of CO_2_, bicarbonate and H^+^ ions, respectively. Thus, as bicarbonate is secreted into the colonic lumen, it combines with H^+^ ions to form CO_2_ and H_2_O to balance the equilibrium equation. This balancing also occurs during CO_2_ secretion and absorption. The pH was then calculated from the concentration of H^+^ ions, where $s_{{H^{ + } }}$ must satisfy both the above equilibrium equation and charge balance.

#### Substrates and metabolites

The influx of substrates and metabolites into the model was limited to the first compartment, with the exception of mucin and bicarbonate. Rates of substrate inflow from dietary sources were taken as equal to those from experimental estimates in the literature (Additional file 1: Sect. 3). Free sulphate from the diet was included at an inflow rate of 0.86 g d^−1^ [59], which was important to the SRB MFG.

In the case of mucin, it was estimated that between 2.7 and 7.3 g d^−1^ is secreted into the colon [59, 60], so 5 g d^−1^ was set as the microPop:Colon influx. Unlike the other metabolites, mucin influx occurred in every compartment, proportionally to the volume of each compartment. For mucin degradation, it was assumed that mucin is made up of mostly carbohydrate, with smaller proportions of protein and sulphate [11, 61]. An analysis of colonic microbial genomes emphasised the capacity of many *Bacteroides* strains to be effective degraders of common mucin structures [62]. *Akkermansia muciniphila* was also implicated as a major mucin degrader, and only this species and certain *Bacteroides* strains were capable of encoding mucin-desulphating sulphatases. Since microPop did not include *A. muciniphila* explicitly and this inclusion has not been made here due to a lack of parameterising data, the Bacteroides MFG was the sole degrader of mucin in the model. The following pathway was selected for the breakdown of mucin by the Bacteroides MFG:

$${1}\;{\text{g}}\;{\text{Mucin}} \to 0.0{5}\;{\text{g}}\;{\text{Sulphate}}\; + \;0.{2}\;{\text{g}}\;{\text{Protein}} + X{\text{g}}\;{\text{NSP}} + \left( {0.{75} - X} \right)\;{\text{g}}\;{\text{Sugars}}$$

where *X* is the unknown weight of mucin carbohydrate that is broken down to NSP rather than simple sugars. It is not clear what proportion of mucin carbohydrate is degraded to simple sugars versus more complex polysaccharides and this likely varies between degrading strains and mucin structures [62]. An approximate value of *X* = 0.5 was assumed. Parameter values for this Bacteroides MFG metabolic pathway were based on the metabolism of chondroitin sulphate and porcine mucin by *Bacteroides thetaiotaomicron* and are listed in Additional file 1: Sect. 5 [63].

Finally, in order to capture the contribution of SCFAs to host nutrition, host absorption of acetate, propionate and butyrate was included. Previous research implied minimal variation between the absorption rates of these three SCFAs in digestive environments [64–66]. Ruppin et al. [66] experimentally tested the absorption rates of varied concentrations of SCFAs perfused into the colon and an absorption rate of approximately 0.4 h^−1^ was calculated from this data, which is applied to acetate, propionate and butyrate in microPop:Colon.

#### MFGs

The eleven MFGs of microPop:Colon were made up of the ten MFGs from the original microPop model (with the aforementioned alterations to the methanogen, acetogen and Bacteroides MFGs), and the novel SRB MFG. Unless stated as changed specifically for microPop:Colon, all values used were those of the original model. For the initial microbial population sizes, 16S rRNA data from faecal samples were used, converted to microPop MFGs according to Kettle et al. [11] (Additional file 1: Sect. 4). This method assigns the faecal 16S rRNA data to microPop MFGs based on the closest match between taxa and MFGs. In instances where there was no clear match between a single probe and MFG, the total 16S count obtained with the probe was split between the relevant MFGs. In instances where no probe corresponded to one of the microPop MFGs (e.g. no methanogen enumerations were made), an assumption was made about the relative proportion of that MFG in the total microbial community, and this value was taken as the initial condition for the model. For the methanogen MFG, this assumption was that the methanogen abundance was equal to 0.1% of the total bacterial abundance (see Additional file 1: Sect. 4). It should also be noted that the initial MFG concentrations from the data of Walker et al. [13] were used as the initial conditions for simulations where other data was not available, including the microPop:Colon simulations. The implications of this assumption are noted in the discussion. No cell death was included in the model, as this was assumed negligible compared to washout, which was included.

### Computation

Although microPop was used as the basis for microPop:Colon, the equations for microPop:Colon were implemented in MATLAB (The MathWorks; www.mathworks.com) rather than R, the format for the original publication, for ease of code management. The availability of microPop functionality in both MATLAB and R should facilitate wider usage of the model. The mathematical structure and parameter values given in the supporting material of the microPop package were used to transition the tool between software. Upon completion, the MATLAB version was tested to ensure that its predictions were consistent with those of the R version. This testing was carried out for the pH static experiment displayed in Fig. 1 and the pH shift experiment displayed in Fig. 2. For all MFGs and metabolites, the predictions of the R and MATLAB versions were identical to four significant figures. Discrepancies beyond this level of accuracy were likely due to the differing numerical solvers used in the computation and were deemed insignificant.

The adaptations of the model to the human colonic environment described above were all carried out in the MATLAB environment. The code is available in the BioModels repository (https://www.ebi.ac.uk/biomodels/MODEL2006210002).

## Supplementary Information

**Additional file 1**: Contains supporting text and tables relevant to the Methods.

**Additional file 2**: Contains additional figures and a table of parameters of model fit.

**Additional file 3**: Contains the model data analysed.

## Data Availability

The model is available in the BioModels repository, https://www.ebi.ac.uk/biomodels/MODEL2006210002. Datasets generated and analysed during the current study are available in the additional files.

## Abbreviations

MFG: Microbial functional group
SRB: Sulphate-reducing bacteria
SCFA: Short chain fatty acid
NSP: Non-starch polysaccharide
NoS, NormS, HighS: Zero sulphate, normal sulphate and high sulphate availability conditions
