# Supplementary material for: Examination of hydrogen cross-feeders using a colonic microbiota model
Source: BMC Bioinformatics. 2021 Jan 6;22:3. doi: 10.1186/s12859-020-03923-6 (PMC7789523; doi:10.1186/s12859-020-03923-6)
Supplement: Supplementary file 1 — Additional file 1: Contains supporting text and tables relevant to the Methods. [file 12859_2020_3923_MOESM1_ESM.docx]

# Additional file 1

## Section 1: microPop equations

The Monod-based equations constructed and solved by microPop are fully described in the supporting materials for the R package [1] and in a previous publication of a similar model structure [2]. Here, we briefly describe the general form of the equations.

Let $X$ denote the concentration (g L^-1^) of a single MFG population. The rate of change of $X$ is given by the differential equation

|  | $\frac{dX}{dt}=X_{in}+\mu X-DX$ | (1.1) |
| --- | --- | --- |

where $X_{in}$ is the inflow of the MFG into the model compartment, $\mu$ is the growth rate of the MFG and $D$ is the dilution rate of the model compartment.

Each MFG has a given number of metabolic pathways available to it, $N_{m}$. The growth rate of the MFG, $\mu$ (h^-1^), is calculated as follows

|  | $\mu=\sum_{i=1}^{N_{m}} \frac{{\mu_{i}}^{2}}{\sum_{j=1}^{N_{m}} \mu_{j}}$ | (1.2) |
| --- | --- | --- |

where $\mu_{i}$ are the growth rates (h^-1^) of the MFG calculated for each pathway available to the MFG. The growth rate of the MFG is therefore scaled by the magnitude of the growth rate for each available pathway, rather than being the sum of all pathway growth rates.

$\mu_{i}$ is calculated differently depending on the nature of the substrates. If a substrate is perfectly substitutable for another on the same pathway, then each substrate is used indiscriminately according to its concentration. Let

|  | $\lambda_{Ss}(S_{i})=\frac{S_{i}/K_{i}}{1+\sum_{j=1}^{N_{s}} S_{j}/K_{j}}$ | (1.3) |
| --- | --- | --- |

where $\lambda_{Ss}$ is the growth limitation function for the substitutable substrate $S_{i}$. $K_{i}$ is the half-saturation constant for $S_{i}$. $K_{j}$ is the half-saturation constant for $S_{j}$, with $j$ representative of all substitutable substrates available on this pathway. $N_{s}$ is the number of substitutable substrates on the pathway.

If a substrate is essential, with no growth possible in its absence, then the growth limitation function is simply:

|  | $\lambda_{Se}\left( S_{i} \right)=\frac{S_{i}}{S_{i}+K_{i}}.$ | (1.4) |
| --- | --- | --- |

The final substrate class is boost substrates: substrates that, if available, increase the growth rate on a certain pathway by a fixed proportion. Let $f_{b}$ be the proportion of the maximum growth rate achievable by the MFG in the absence of the boost substrate. Then:

|  | $\lambda_{b}=f_{b}+\left( 1-f_{b} \right)\lambda_{Se}\left( S_{b} \right).$ | (1.5) |
| --- | --- | --- |

where $\lambda_{b}$ is the boost limitation function. Finally, pH limitation is included as the value $\lambda_{pH}$, lying in the interval [0,1], determined by the pH preferences of the MFG and the current pH value in the model compartment. Each MFG has four pH corners which define its upper and lower bounds for growth, and the upper and lower bounds for optimal growth. A $\lambda_{pH}$ value of 1 is assigned if the pH is within the optimal growth bounds and a value of 0 is assigned if the pH is outside the growth bounds. If the pH is outside the optimal growth bounds but inside the growth bounds, a $\lambda_{pH}$ value between 0 and 1 is assigned, with this value linearly decreasing as pH moves further from the optimal range.

The general form of$\mu_{i}$ is thus:

|  | $\mu_{i}=\lambda_{pH}\lambda_{b}\left( \mu_{ess}^{m}\prod_{j=1}^{N_{ess}} \lambda_{Se}\left( S_{j} \right) \right)\left( \sum_{l=1}^{N_{s}} \mu_{l}^{m}\lambda_{Ss}(S_{l}) \right)$ | (1.6) |
| --- | --- | --- |

where $N_{ess}$ is the number of essential resources on the pathway. If there exist solely substitutable resources on the pathway, then the terms relating to essential substrate metabolism are removed, but otherwise $\mu_{ess}^{m}$ is the maximum growth rate of this MFG on the essential pathway.$\mu_{l}^{m}$ is the maximum growth rate of the MFG on substitutable resource $S_{l}$.

The rate of change in the concentration of a resource is the sum of its inflow into the model compartment and its production by MFGs, minus its outflow from the model compartment and uptake by MFGs. The uptake rate of a substitutable resource $S_{i}$ by MFG $X$ on the pathway with growth rate $\mu_{i}$ is:

|  | $\lambda_{pH}\lambda_{b}\lambda_{Ss}\left( S_{i} \right)\mu_{i}^{m}\frac{X}{Y_{X,S_{i}}}$ | (1.7) |
| --- | --- | --- |

where $Y_{X,S_{i}}$ is the growth yield of MFG $X$ on substrate $S_{i}$. In the case of essential resources, where multiple resources are considered, the stoichiometry of the reaction is included in the uptake. Let $S_{k}$ be the key resource of the pathway (one of the essential resources). Then the uptake rate of an essential resource $S_{i}$ is:

|  | ${\left( \frac{m_{i}n_{i}}{m_{k}n_{k}} \right)\lambda}_{pH}\lambda_{b}\mu_{ess}^{m}\lambda_{Se}\left( S_{k} \right)\frac{X}{Y_{X,S_{k}}}.$ | (1.8) |
| --- | --- | --- |

Here, the values $m_{i}$ and $n_{i}$ are the molar mass and reaction stoichiometry of $S_{i}$, and $m_{k}$ and $n_{k}$ the corresponding values for the key resource.

Finally, for the uptake of a boost substrate, we take the uptake of the substitutable resources and scale this by the reaction stoichiometry for the boost substrate to give the boost substrate uptake rate:

|  | $\frac{m_{b}n_{b}}{\frac{1}{N_{s}}\sum_{i=1}^{N_{s}} m_{i}n_{i}}\left( \lambda_{pH}\lambda_{b}\sum_{j=1}^{N_{s}} \left( \frac{\lambda_{Ss}\left( S_{j} \right)\mu_{j}^{m}}{Y_{X,S_{j}}} \right)X \right).$ | (1.9) |
| --- | --- | --- |

Here, $i$ indexes the substitutable substrates involved in the pathway, and $b$ indexes the boost substrate.

For the production of metabolites by MFGs, let $U_{i}$ denote the uptake of $S_{i}$ on a given pathway. Then the production of metabolite $j$ is given by:

|  | $\frac{m_{j}n_{j}}{\sum_{k=1}^{N_{p}} m_{k}n_{k}}\left( \sum_{i=1}^{N_{alls}} U_{i}-dX \right)$ | (1.10) |
| --- | --- | --- |

where $N_{p}$ and $N_{alls}$ are the total number of products and substrates involved in the pathway, respectively, and $dX$ is the growth rate of the MFG on this pathway.

## Section 2: Summary of parameter values and metabolic pathways for the three hydrogenotrophic MFGs

The metabolic pathways available to the Acetogen MFG are as follows:

4 H_2_ + 2 CO_2_ → Acetate + 2 H_2_O

NSP/RS/Sugars + 2 Formate → 3 Acetate + 2 H_2_ + 2 CO_2_

NSP/RS/Sugars → 3 Acetate.

The metabolic pathways available to the Methanogen MFG are as follows:

4 H_2_ + CO_2_ → CH_4_ + 2 H_2_O

4 Formate → CH_4_ + 2 H_2_O + 3 CO_2_.

The metabolic pathways available to the SRB MFG are as follows:

5 H_2_ + 1 Sulphate → H_2_S + 4 H_2_O

4 Formate + Sulphate → H_2_S + 4 Bicarbonate + H_2_

Lactate + 2 H_2_O → Acetate + 2.5 H_2_ + Bicarbonate.

The SRB metabolic pathway involving formate was not studied by Smith et al. [3] and there is a lack of data to parameterise a monoculture model for this pathway. Therefore, the maximum growth rate and yield parameters for formate utilisation are taken as equal to those of hydrogen metabolism, while the half-saturation value for formate is taken as equal to that of the acetogens and methanogens from microPop.

| **Table S2.1**. Newly derived parameter values for the hydrogenotrophic MFGs. Parameter value is given, followed by the substrate to which it corresponds in brackets. Values in bold are alterations or additions to the original microPop values; all others are unchanged. | | | | |
| --- | --- | --- | --- | --- |
|  |  | Acetogens | Methanogens | SRB |
| Pathway 1 | halfSat (g L^-1^) | **0.5975 (H_2_)**  0.001 (CO_2_) | **0.0214 (H_2_)**  0.001 (CO_2_) | **2.02 x10^-6^ (H_2_)**  **0.00307 (Sulphate)** |
|  | yield (g g^-1^) | **0.9218 (H_2_)** | **0.891 (H_2_)** | **0.0463 (Sulphate)** |
|  | maxGrowthRate (d^-1^) | **9.55** | **2.5** | **0.72** |
| Pathway 2 | halfSat (g L^-1^) | 0.001 (Formate)  0.001 (NSP/RS/Sugars) | 0.001 (Formate) | **0.001 (Formate)**  **0.00307 (Sulphate)** |
|  | yield (g g^-1^) | 0.286/0.333/0.333 (NSP/RS/Sugars) | 0.00724 (Formate) | **0.0463 (Sulphate)** |
|  | maxGrowthRate (d^-1^) | 6/6/24 (NSP/RS/Sugars) | 2.4 | **0.72** |
| Pathway 3 | halfSat (g L^-1^) | 0.001 (NSP/RS/Sugars) | - | **0.126 (Lactate)** |
|  | yield (g g^-1^) | 0.286/0.333/0.333 (NSP/RS/Sugars) | - | **0.0627 (Lactate)** |
|  | maxGrowthRate (d^-1^) | 6/6/24 (NSP/RS/Sugars) | - | **2.784** |

## Section 3: Metabolite influx rates for comparison to experimental data

Metabolite influx values were converted from the media contents lists in the original publications to microPop metabolites following Kettle et al. [2]. The conversion for the growth medium in Walker et al. [4] is shown in Tables S3.1 and S3.2. The medium of Belenguer et al. [5] is very similar to that of Walker et al. [4], except for protein influx, which was 2 g L^-1^ d^-1^. The conversion for the growth medium of Payne et al. [6] is shown in Tables S3.3 and S3.4.

For the simulation of the human colon, microPop:Colon influx concentrations are given in Table S3.5.

| **Table S3.1.** Conversion of Walker et al. [4] growth medium constituents to microPop metabolites. | | |
| --- | --- | --- |
| Ingredient | Concentration (% wt/vol) | Conversion factors |
| Potato starch | 0.5 | 100% RS |
| Xylan | 0.06 | 100% NSP |
| Pectin | 0.06 | 100% NSP |
| Amylopectin | 0.06 | 100% RS |
| Arabinogalactan | 0.06 | 100% NSP |
| Peptide mixture | 0.1 or 0.6* | 100% Protein |
| *Varied between experiments | | |

| **Table S3.2.** microPop metabolite inflow rates, based on Walker et al. [4]. Initial concentration and inflow rate for all other metabolites are set to 0. | | |
| --- | --- | --- |
| **Metabolite** | **Initial concentration (g L^-1^)** | **Inflow rate (g L^-1^ d^-1^)** |
| Protein | 1 or 6* | 1 or 6* |
| NSP | 1.8 | 1.8 |
| RS | 5.6 | 5.6 |
| Acetate | 1.95 | 0 |
| Propionate | 0.67 | 0 |
| Buytrate | 0.44 | 0 |
| CO_2_ | 10 | 10 |
| H_2_O | 10 | 10 |
| *Varied between experiments | | |

| **Table S3.3.** Conversion of Payne et al. [7] growth medium constituents to microPop metabolites. | | | | |
| --- | --- | --- | --- | --- |
| **Ingredient** | **Concentration (g L^-1^)** | | | **Conversion factors** |
|  | **HE medium** | **NE medium** | **LE medium** |  |
| Pectin | 1 | 2 | 1.2 | 100% NSP |
| Xylan | 1 | 2 | 1.2 | 100% NSP |
| Arabinogalactan | 2 | 2 | 1.2 | 100% NSP |
| Guar gum | 1 | 1 | 0.4 | 100% NSP |
| Inulin | 1 | 1 | 0.4 | 100% NSP |
| Soluble starch | 9 | 5 | 2.4 | 100% RS |
| Granular amylopectin maize starch | 4 | 4 | 1 | 100% RS |
| D-fructose | 6 | 3 | 1.2 | 100% Sugars |
| Mucin | 4 | 4 | 4 | 100% Mucin |
| Casein acid hydrolysate | 5 | 3 | 1.2 | 100% Protein |
| Peptone water | 5 | 5 | 2 | 100% Protein |
| Bacto^TM^ Tryptone | 5 | 5 | 2 | 100% Protein |
| Yeast extract | 4.5 | 4.5 | 1.8 | 100% Protein |

| **Table S3.4.** microPop metabolite concentrations in medium, based on Payne et al. [7]. Concentrations for all other metabolites are set to 0. | | | |
| --- | --- | --- | --- |
| **Metabolite** | **HE medium (g L^-1^)** | **NE medium (g L^-1^)** | **LE medium (g L^-1^)** |
| Protein | 19.5 | 17.5 | 7 |
| NSP | 6 | 8 | 4.4 |
| RS | 13 | 9 | 3.4 |
| Sugars | 6 | 3 | 1.2 |
| Mucin | 4 | 4 | 4 |
| CO_2_ | 10 | 10 | 10 |
| H_2_O | 10 | 10 | 10 |

| **Table S3.5.** microPop:Colon metabolite initial concentrations and inflow rates. | | | |
| --- | --- | --- | --- |
| **Metabolite** | **Initial concentration in proximal compartment (g L^-1^)** | **Inflow rate (g L^-1^ d^-1^)** | **Reference/explanation** |
| Protein | 5 | 5 | [8] |
| NSP | 12 | 12 | [8] |
| RS | 20 | 20 | [8] |
| Sugars | 0 | 0 | Simple sugars assumed to have been absorbed in small intestine |
| H_2_ | 0 | 0 | ^c^ |
| CO_2_ | 0.22^b^ | 0 |  |
| CH_4_ | 0 | 0 | ^c^ |
| H_2_0 | 10 | 10 | Set high as assumed abundant and non-limiting in the colon environment |
| Acetate | 0 | 0 | ^c^ |
| Propionate | 0 | 0 | ^c^ |
| Succinate | 0 | 0 | ^c^ |
| Lactate | 0 | 0 | ^c^ |
| Formate | 0 | 0 | ^c^ |
| Ethanol | 0 | 0 | ^c^ |
| Butyrate | 0 | 0 | ^c^ |
| Sulphate | 0.86 | 0.86 | [9] |
| H_2_S | 0 | 0 | ^c^ |
| Bicarbonate | 1.53^b^ | 13.6 (g d^-1^)^a^ | [10-12] |
| Other | 0 | 0 | ^c^ |
| Mucin | 5^a^ | 5^a^ | [9, 13] |
| ^a^Divided into each of the three compartments based on their relative volume in the discrete model.  ^b^CO_2_ and bicarbonate concentrations were set initially at these values in all model compartments. The initial CO_2_ concentration was calculated based on the bicarbonate value to ensure neutral pH at model initiation.  ^c^Product of microbial metabolism so assumed negligible before colon. | | | |

## Section 4: Microbial abundance calculations

The probe data from Walker et al. [4] and Payne et al. [7] was used to derive initial MFG abundances as performed by Kettle et al. [2]. The conversion used was:

Concentration (g L^-1^) = [Counts (cells ml^-1^)] x [8.5 x 10^-9^]

with the assignment of probe data to MFGs shown in Tables S4.1 and S4.3 for the Walker et al. [4] and Payne et al. [6] datasets, respectively. Since no data was available for the SRB from Walker et al. [4], the initial count for this MFG was assumed equal to that of the methanogens. Conversions from the original publication to microPop MFGs for all data used in the validations is shown in Tables S4.2 and S4.4.

For the simulation of the experimental work carried out by Payne et al. [7], an initial inoculum concentration of 1 g L^-1^ was assumed. This was made up of all 11 MFGs, with the concentration of each determined by its proportion of the initial population, as calculated from Table S4.4.

| **Table S4.1.** Assignment of probe counts from Walker et al. [4] to microPop MFGs | |
| --- | --- |
| **MFG** | **Probe assignment** |
| Bacteroides | Bac303 |
| NoButyStarchDeg | 0.5 (Rfla729+Rbro730) + 0.3 (Erec482-Rrec584) |
| NoButyFibreDeg | 0.5 (Rfla729+Rbro730) + 0.3 (Erec482-Rrec584) |
| LactateProducers | Bif164 + Ato291 |
| ButyrateProducers1 | Rrec584 |
| ButyrateProducers2 | Fprau645 |
| PropionateProducers | Prop853 |
| ButyrateProducers3 | 0.3 (Erec482 - Rrec584) |
| Acetogens | 0.1 (Erec482 - Rrec584) |
| Methanogens | 0.001 Eub |
| SRB | 0.001 Eub |

| **Table S4.2.** MFG concentrations converted from original measurements in Walker et al. [4]. | | | | | | | | | | |
| --- | --- | --- | --- | --- | --- | --- | --- | --- | --- | --- |
| **MFG** | **Concentration (g L^-1^)** | | | | | | | | | |
|  | **Figure 1 (6 g L^-1^ peptide, Donor 2)** | | **Additional figure 1 (1 g L^-1^ peptide, Donor 2)** | | **Additional figure 2 (6 g L^-1^ peptide, Donor 1)** | | | **Figure 2 (6 g L^-1^ peptide, Donor 2)** | | |
|  | **Initial** | **Final** | **Initial** | **Final** | **Initial** | **Mid-point** | **Final** | **Initial** | **Mid-point** | **Final** |
| Bacteroides | 0.0399 | 1.26 | 0.0399 | 0.0629 | 0.0179 | 0.349 | 1.879 | 0.0527 | 0.214 | 2.35 |
| NoButyStarchDeg | 0.000255 | 0.0464 | 0.000255 | 0.0293 | 0.0328 | 0.0928 | 0.0553 | 0.0396 | 0.125 | 0.0966 |
| NoButyFibreDeg | 0.000255 | 0.0464 | 0.000255 | 0.0293 | 0.0328 | 0.0928 | 0.0553 | 0.0396 | 0.125 | 0.0966 |
| LactateProducers | 0.0468 | 0.0068 | 0.0468 | 0.0119 | 0.00425 | 0.0128 | 0.00255 | 0.0196 | BD | BD |
| ButyrateProducers1 | 0.0289 | BD | 0.0289 | 0.0153 | 0.0213 | 0.205 | BD | 0.0595 | 0.29 | BD |
| ButyrateProducers2 | 0.00255 | 0.0017 | 0.00255 | BD | 0.0187 | 0.085 | 0.0272 | 0.0485 | 0.0697 | 0.0519 |
| PropionateProducers | 0.0332 | 0.00765 | 0.0332 | 0.00085 | 0.0111 | 0.0153 | 0.111 | 0.0315 | 0.0476 | 0.0536 |
| ButyrateProducers3 | 0.000255 | 0.0464 | 0.000255 | 0.0293 | 0.0247 | 0.0928 | 0.0362 | 0.0337 | 0.125 | 0.0966 |
| Acetogens | 8.5e-05 | 0.0155 | 8.5e-05 | 0.00978 | 0.00825 | 0.0309 | 0.0121 | 0.0112 | 0.0417 | 0.0322 |
| Methanogens | 0.000232 | 0.00164 | 0.000232 | 0.000388 | 0.000428 | 0.00109 | 0.00224 | 0.00041 | 0.00207 | 0.0029 |
| SRB | 0.000232 | ND | 0.000232 | ND | 0.000428 | ND | ND | 0.00041 | ND | ND |
| Note that the inocula for Figures 1 &2 are identical, as both these experiments used a microbial population from the same faecal sample.  ND: No Data. BD: Below Detection. | | | | | | | | | | |

| **Table S4.3.** Assignment of bacterial populations from Payne et al. [7] to microPop MFGs | |
| --- | --- |
| **microPop MFG** | **Assignment from measured bacterial groups in original publication (primers used)** |
| Bacteroides | *Bacteroides* (Bac303F, Bfr-Femrev) |
| NoButyStarchDeg | 0.5 * [Firmicutes (Firm934F, Firm1060R) - *Roseburia* sp., *E. rectale* (RrecF, Rrec630mR) - *E. hallii* (EhalF, EhalR) - *F. prausnitzii* (FPR-2F, Fprau645R) - *Veillonella* spp. (Vpa-X84005_F, Vpa_X84005_R) - *Lactobacillus* (F_Lacto 05, R_Lacto 04)] |
| NoButyFibreDeg | 0.5 * [Firmicutes (Firm934F, Firm1060R) - *Roseburia* sp., *E. rectale* (RrecF, Rrec630mR) - *E. hallii* (EhalF, EhalR) - *F. prausnitzii* (FPR-2F, Fprau645R) - *Veillonella* spp. (Vpa-X84005_F, Vpa_X84005_R) - *Lactobacillus* (F_Lacto 05, R_Lacto 04)] |
| LactateProducers | *Bifidobacterium* (xfp-fw, xfp-rv) + *Lactobacillus* (F_Lacto 05, R_Lacto 04) + Enterobacteriaceae (Eco1457F, Eco1652R) |
| ButyrateProducers1 | *Roseburia* sp., *E. rectale* (RrecF, Rrec630mR) |
| ButyrateProducers2 | *F. prausnitzii* (FPR-2F, Fprau645R) |
| PropionateProducers | *Veillonella* spp. (Vpa-X84005_F, Vpa_X84005_R) |
| ButyrateProducers3 | *E. hallii* (EhalF, EhalR) |
| Acetogens | 0.01 * Firmicutes (Firm934F, Firm1060R) |
| Methanogens | 0.001 * Total bacteria (Eub 338F, Eub 518R) |
| SRB | Sulfate-reducing bacteria (dsrA_290F, dsrA_660R) |

| **Table S4.4.** MFG concentrations converted from original measurements in Payne et al. [7]. | | | | | | | | | | | | | | | | | | | | |
| --- | --- | --- | --- | --- | --- | --- | --- | --- | --- | --- | --- | --- | --- | --- | --- | --- | --- | --- | --- | --- |
| **MFG** | **Concentration (g L^-1^)** | | | | | | | | | | | | | | | | | | | |
|  | **Faeces** | | **High-energy** | | | | | | **Normal-energy** | | | | | | **Low-energy** | | | | | |
|  |  | | **Comp. 1** | | **Comp. R2** | | **Comp. R3** | | **Comp. 1** | | **Comp. R2** | | **Comp. R3** | | **Comp. 1** | | **Comp. R2** | | **Comp. R3** | |
|  | **A** | **B** | **A** | **B** | **A** | **B** | **A** | **B** | **A** | **B** | **A** | **B** | **A** | **B** | **A** | **B** | **A** | **B** | **A** | **B** |
| Bacteroides | 4.26E-03 | 3.83E-03 | 1.07E-06 | 5.36E-05 | 1.07E-02 | 6.75E-03 | 1.35E-02 | 8.50E-03 | 3.38E-05 | 2.14E-04 | 2.14E-02 | 6.75E-03 | 2.14E-02 | 8.50E-03 | 2.14E-03 | 4.26E-03 | 1.07E-02 | 1.35E-02 | 2.14E-02 | 1.35E-02 |
| NoButyStarchDeg | 5.98E-04 | 2.13E-03 | 2.61E-03 | 2.00E-03 | 3.08E-03 | 2.78E-03 | 3.14E-03 | 1.42E-03 | 0 | 2.61E-03 | 3.50E-04 | 2.86E-03 | 6.37E-04 | 2.49E-03 | 1.15E-02 | 1.00E-02 | 3.65E-03 | 4.88E-03 | 3.83E-04 | 3.99E-03 |
| NoButyFibreDeg | 5.98E-04 | 2.13E-03 | 2.61E-03 | 2.00E-03 | 3.08E-03 | 2.78E-03 | 3.14E-03 | 1.42E-03 | 0 | 2.61E-03 | 3.50E-04 | 2.86E-03 | 6.37E-04 | 2.49E-03 | 1.15E-02 | 1.00E-02 | 3.65E-03 | 4.88E-03 | 3.83E-04 | 3.99E-03 |
| LactateProducers | 1.71E-05 | 1.34E-04 | 2.69E-03 | 4.85E-04 | 3.03E-03 | 2.16E-03 | 2.81E-03 | 1.82E-03 | 4.31E-04 | 1.79E-03 | 2.23E-03 | 1.62E-03 | 4.73E-03 | 1.76E-03 | 1.05E-04 | 1.91E-03 | 1.81E-03 | 1.48E-03 | 3.72E-03 | 7.39E-04 |
| ButyrateProducers1 | 5.36E-03 | 2.14E-04 | 3.38E-05 | 2.69E-03 | 2.69E-05 | 4.26E-03 | 1.70E-05 | 8.50E-04 | 5.36E-03 | 1.07E-03 | 8.50E-04 | 1.07E-03 | 6.75E-04 | 6.75E-04 | 1.07E-02 | 1.35E-02 | 2.69E-03 | 1.70E-02 | 1.07E-03 | 5.36E-03 |
| ButyrateProducers2 | 1.07E-03 | 8.50E-04 | 5.36E-08 | 5.36E-09 | 3.38E-05 | 1.07E-05 | 3.38E-05 | 1.35E-05 | 8.50E-08 | 4.26E-09 | 1.07E-05 | 8.50E-06 | 1.70E-05 | 1.07E-05 | 3.38E-08 | 4.26E-08 | 8.50E-06 | 3.38E-06 | 1.70E-05 | 2.69E-06 |
| PropionateProducers | 8.50E-04 | 8.50E-13 | 8.50E-13 | 8.50E-13 | 1.07E-09 | 8.50E-13 | 8.50E-10 | 8.50E-13 | 8.50E-13 | 8.50E-13 | 2.14E-09 | 8.50E-13 | 8.50E-13 | 8.50E-13 | 8.50E-08 | 8.50E-13 | 5.36E-08 | 8.50E-13 | 8.50E-13 | 8.50E-13 |
| ButyrateProducers3 | 2.14E-05 | 1.07E-05 | 1.07E-04 | 6.75E-05 | 5.36E-04 | 8.50E-04 | 4.26E-04 | 5.36E-04 | 2.14E-05 | 4.26E-04 | 1.35E-04 | 1.70E-03 | 1.70E-04 | 1.07E-03 | 2.14E-05 | 1.07E-04 | 3.38E-05 | 8.50E-05 | 1.35E-05 | 6.75E-05 |
| Acetogens | 8.50E-05 | 5.36E-05 | 5.36E-05 | 6.75E-05 | 6.75E-05 | 1.07E-04 | 6.75E-05 | 4.26E-05 | 5.36E-05 | 6.75E-05 | 1.70E-05 | 8.50E-05 | 2.14E-05 | 6.75E-05 | 3.38E-04 | 3.38E-04 | 1.07E-04 | 2.69E-04 | 2.14E-05 | 1.35E-04 |
| Methanogens | 8.50E-05 | 1.07E-04 | 1.07E-04 | 1.07E-04 | 1.70E-04 | 1.70E-04 | 1.70E-04 | 1.35E-04 | 6.75E-14 | 1.07E-04 | 1.35E-04 | 1.07E-04 | 1.70E-04 | 1.07E-04 | 1.70E-05 | 3.38E-05 | 2.69E-05 | 4.26E-05 | 1.70E-05 | 3.38E-05 |
| SRB | 3.38E-07 | 3.38E-06 | 4.26E-09 | 4.26E-09 | 1.07E-09 | 1.07E-09 | 2.69E-09 | 2.69E-09 | 5.36E-09 | 3.38E-09 | 1.07E-09 | 4.26E-09 | 2.69E-09 | 1.35E-09 | 1.07E-09 | 1.35E-09 | 1.07E-08 | 2.69E-09 | 2.69E-06 | 8.50E-06 |
| Comp. = Compartment | | | | | | | | | | | | | | | | | | | | |

## Section 5: Addition of mucin and alterations to Bacteroides MFG

We added mucin as a metabolite in microPop, assuming a molar mass of 162.12 g mol^-1^ so that the metabolic pathway we formulate maintains mass balance. We would expect mucin to have a greater molar mass than this, but this simplification has no effect on the model output.

We added the following metabolic pathway to the Bacteroides MFG, based on literature evidence and previous modelling [2, 14]:

Mucin → 0.2 Protein + 0.5 NSP + 0.25 Sugars + 0.05 Sulphate.

Since we were unable to obtain useable data to parameterise the growth of a *Bacteroides* strain on mucin, we have made assumptions based on the literature for the parameter values corresponding to this metabolic pathway. The half-saturation constant was set to 0.001 g L^-1^, to match that of Protein, NSP and RS for the Bacteroides MFG. The yield was set to 0.01 g g^-1^, as we expect this MFG to grow predominantly during the subsequent metabolism of mucin constituent metabolites, rather than during mucin breakdown itself. The maximum growth rate was set to 3.3 d^-1^, based on growth rates of *Bacteroides thetaiotaomicron*, an abundant GIT strain, cultured with porcine mucin [15]. Finally, we altered the pH preferences of the Bacteroides MFG, so that this MFG is now active down to a lower limit of pH 5.3, based on experimental evidence [16].

## Section 6: pH modelling

As mentioned in the main text, the pH is determined from the concentration of H^+^ ions, which must satisfy both the carbonate acid-base equilibrium equation and the charge balance equation. The charge balance equation is as follows:

|  | $s_{{cat}^{+}}+s_{H^{+}}-\left( s_{HCO_{3}^{-}}+s_{SCFA^{-}}+s_{OH^{-}}+C \right)=0$ | (S6.1) |
| --- | --- | --- |

where $s_{i}$ denotes the molar concentration of molecule $i$, and $i$ may represent ${cat}^{+}$ (miscellaneous cations), $H^{+}$ (H^+^ ions), $HCO_{3}^{-}$ (bicarbonate), $SCFA^{-}$ (the short-chain fatty acids) or $OH^{-}$ (hydroxide ions). $C$ is the number of moles of bicarbonate produced from CO_2_ during buffering of the intestinal lumen. The value of $C$ is determined via the carbonate acid-base equilibrium equation, as discussed later in this section. Note that this value is negative if the net reaction is conversion of bicarbonate and H^+^ ions to CO_2_.

We next define acid-base equilibrium equations for SCFA and hydroxide:

|  | $s_{{SCFA}^{-}}=\frac{K_{SCFA}s_{SCFA}}{K_{SCFA}+s_{H^{+}}}$ | (S6.2) |
| --- | --- | --- |
|  | $s_{{OH}^{-}}=\frac{K_{w}}{s_{H^{+}}}$ | (S6.3) |

where $K_{SCFA}$ and $K_{w}$ are the acid-base equilibrium constants for SCFA and water, respectively. $s_{SCFA}$ is the total concentration of both dissociated and undissociated SCFA:

$$s_{SCFA}=s_{SCFA^{-}}+s_{HSCFA}.$$

Using these acid-base equilibrium equations, we may rewrite equation S6.1 as:

|  | $s_{{cat}^{+}}+s_{H^{+}}-\left( s_{HCO_{3}^{-}}+\frac{K_{SCFA}s_{SCFA}}{K_{SCFA}+s_{H^{+}}}+\frac{K_{w}}{s_{H^{+}}}+C \right)=0.$ | (S6.4) |
| --- | --- | --- |

Equation S6.4 can be rearranged to give a degree three polynomial in $s_{H^{+}}$, which is then solved by the model and used to calculate the pH value:

$${\mathrm{pH}=-log(s}_{H^{+}}).$$

Note that the use of the charge balance equation and acid-base equilibrium equations that form the basis of this pH model are adapted from Batstone et al. [17] and Muñoz-Tamayo et al. [18]. To capture the interconversion between CO_2_ + H_2_O and HCO_3_^-^ + H^+^, we append the following term to the microPop:Colon differential equation for bicarbonate:

$$r[\mathrm{HCO}_{3}^{-}]({K_{a,CO_{2}}s_{CO2}-s}_{HCO_{3}^{-}}s_{H^{+}})$$

where $[\mathrm{HCO}_{3}^{-}]$ is the concentration of bicarbonate in g L^-1^ and $r$ is a rate term with units M^-2^ d^-1^, which is set at a large value, given that the acid-base reaction it controls is assumed to take place almost instantaneously. This term is positive if the equilibrium between CO_2_ and bicarbonate is unbalanced by too great a concentration of CO_2_, and negative if unbalanced by too great a concentration of bicarbonate. The differential equation in $C$ given by:

$$\frac{dC}{dt}=rs_{HCO_{3}^{-}}({K_{a,CO_{2}}s_{CO2}-s}_{HCO_{3}^{-}}s_{H^{+}})$$

gives us a record of the amount of bicarbonate converted to CO2 or vice versa throughout the model simulation. It is this value that can be input into equation S6.4 for determining $s_{H^{+}}$.

The use of this term influences metabolite concentrations towards the balancing of the charge balance equation over very short time periods, far less than a single model timestep. A corresponding term is included in the differential equation for water and CO_2_, with consideration of molar masses as per the normal stoichiometric rules for microPop.

Finally, before the model is initiated, the value of $s_{{cat}^{+}}$ and the initial CO_2_ concentration are calculated to ensure that pH is neutral at initial time. The acid-base equilibrium equation for carbonates can be rearranged to give the initial CO_2_ concentration:

$$s_{CO2}=\frac{{10}^{-7}s_{HCO_{3}^{-}}}{K_{a,CO_{2}}}$$

noting that pH 7 is achieved when $s_{H^{+}}={10}^{-7}$. Thus, the value of $s_{{cat}^{+}}$ remains fixed at:

$$s_{{cat}^{+}}=-s_{H^{+}}+s_{HCO_{3}^{-}}+s_{SCFA^{-}}+s_{OH^{-}}+C={-10}^{-7}+s_{HCO_{3}^{-}}+\frac{K_{SCFA}s_{SCFA}}{K_{SCFA}+{10}^{-7}}+\frac{K_{w}}{{10}^{-7}}+0$$

where only the initial bicarbonate and SCFA concentration may vary between model runs.

Data in the literature for the absorption and secretion rates of CO_2 ­_and bicarbonate in the colon is limited. However, it is known that colonic CO_2_ absorption is rapid, hence its preferred use in medical insufflation [19, 20], and that the CO_2_ concentration in the canine colon tended to an equilibrium value of approximately 75 mM at a maximum rate (using the concentrations tested) of approximately 60 mM h^-1^ [21]. While these values cannot be directly translated to the human colon, an upper estimate absorption coefficient of 65 d^-1^ was assumed in the model to allow for a rapid return to the initial concentration following perturbation by microbially produced CO_2_. Thus, the following term was appended to the differential equation for CO_2_:

$$-\frac{{abs}_{CO2}}{s_{CO2}}(s_{CO2}-s_{CO2,initial})$$

where ${abs}_{CO2}$ is the absorption coefficient and $s_{CO2,initial}$ is the initial CO_2_ concentration.

## Section 7: Parameter values for the Beta parameter set

The alterations to the MFG parameter values in the original microPop MFGs were made by Wang et al. [22] and used in the construction of the Beta parameter set are listed in Table S7.1. Note that only those parameter values that are changed are listed here; all other parameters retain the Alpha parameter set value. Moreover, the parameter changes made to the hydrogenotrophic MFGs and the Bacteroides MFG detailed in Table S2.1 and section S5 supersede those detailed below.

In addition to these MFG parameter changes, the molar mass of ‘Protein’ was changed from 134 to 111 g mol-1 and the molar mass of ‘other’ was changed from 41 to 1 g mol-1. Both of these metabolites are only involved in pathway 2 of the Bacteroides MFG.

| **Table S7.1.** Alterations to MFG parameter values between Alpha and Beta parameter sets | | | |
| --- | --- | --- | --- |
| **MFG** | **Parameter** | **Alpha parameter set values** | **Beta parameter set values** |
| Bacteroides | Maximum growth rates on pathway 1 | NSP: 12  RS: 24 | NSP: 16  RS: 16 |
|  | Maximum growth rates on pathway 2 | Protein: 24 | Protein: 9 |
|  | Stoichiometry on pathway 2 | 3 Protein → 2 Acetate + 1 Propionate + 1 Succinate + 2 H2 + 1 CO2 + 1 other | 6 Protein → 2 Acetate + 1 Propionate + 1 Succinate + 2 H2 + 1 CO2 + 305.68 other |
|  | pH corners | [5.6; 6.35; 7.85; 8.6] | [5.05; 5.8; 7.2; 7.5] |
| NoButyStarchDeg | Maximum growth rates on pathway 1 | NSP: 3.6  RS: 14.4 | NSP: 3  RS: 13 |
|  | pH corners | [5.35; 6.1; 7.6; 8.35] | [4.75; 5.5; 7.2; 7.5] |
| NoButyFibreDeg | Maximum growth rates on pathway 1 | NSP: 16.8  RS: 3.6 | NSP: 16  RS: 6 |
|  | pH corners | [5; 5.75; 7.25; 8] | [4.75; 5.5; 7.2; 7.5] |
| LactateProducers | Maximum growth rates on pathway 1 | NSP: 7.2  RS: 6 | NSP: 9  RS: 11 |
|  | pH corners | [4.95; 5.7; 7.2; 7.95] | [4.5; 5.25; 7.2; 7.5] |
| ButyrateProducers1 | Maximum growth rates on pathway 1 | NSP: 8.4  RS: 8.4 | NSP: 9  RS: 12 |
|  | Stoichiometry on pathway 1 | 2 Hexose (+ 2 Acetate boost) → 3 Butyrate + 2 H2 + 4 CO2 + 2 H2O | 4 Hexose (+ 2 Acetate boost) → 5 Butyrate + 6 H2 + 8 CO2 + 2 H2O |
|  | pH corners | [4.95; 5.7; 7.2; 7.95] | [4.75; 5.5; 7.2; 7.5] |
| ButyrateProducers2 | Maximum growth rates on pathway 1 | NSP: 14.4  RS: 7.2 | NSP: 12  RS: 10 |
|  | pH corners | [4.85; 5.6; 7.1; 7.85] | [4.75; 5.5; 7.2; 7.5] |
| PropionateProducers | Maximum growth rates on pathway 1 | NSP: 7.2  RS: 7.2 | NSP: 6  RS: 6 |
|  | Maximum growth rates on pathway 2 | Lactate: 4.8 | Lactate: 3 |
|  | pH corners | [4.75; 5.5; 7; 7.75] | [4.75; 5.5; 7.2; 7.5] |
| ButyrateProducers3 | Maximum growth rates on pathway 1 | NSP: 7.2  RS: 7.2 | NSP: 6  RS: 6 |
|  | Maximum growth rates on pathway 2 | Lactate: 4.8 | Lactate: 3 |
|  | pH corners | [4.85; 5.6; 7.1; 7.85] | [4.75; 5.5; 7.2; 7.5] |
| Acetogens | Maximum growth rates on pathway 1 | NSP: 7.2  RS: 7.2 | NSP: 6  RS: 6 |
|  | Maximum growth rates on pathway 3 | NSP: 7.2  RS: 7.2 | NSP: 6  RS: 6 |
|  | pH corners | [5.25, 6, 7.5, 8.25] | [4.75, 5.5, 7.2, 7.5] |
| Methanogens | pH corners | [5.25; 6; 7.5; 8.25] | [5.05; 5.8; 7.2; 7.5] |

# References

1. Kettle H, Holtrop G, Louis P, Flint HJ: **microPop: Modelling microbial populations and communities in R**. *Methods Ecol Evol* 2017, **9**:399–409.

2. Kettle H, Louis P, Holtrop G, Duncan SH, Flint HJ: **Modelling the emergent dynamics and major metabolites of the human colonic microbiota**. *Environ Microbiol* 2015, **17**(5):1615-1630.

3. Smith NW, Shorten PR, Altermann E, Roy NC, McNabb WC: **A Mathematical Model for the Hydrogenotrophic Metabolism of Sulphate-Reducing Bacteria**. *Front Microbiol* 2019, **10**(1652).

4. Walker AW, Duncan SH, Carol McWilliam Leitch E, Child MW, Flint HJ: **pH and peptide supply can radically alter bacterial populations and short-chain fatty acid ratios within microbial communities from the human colon**. *Appl Environ Microbiol* 2005, **71**(7):3692-3700.

5. Belenguer A, Holtrop G, Duncan SH, Anderson SE, Calder AG, Flint HJ, Lobley GE: **Rates of production and utilization of lactate by microbial communities from the human colon**. *FEMS Microbiol Ecol* 2011, **77**(1):107-119.

6. Payne AN, Chassard C, Zimmermann M, Müller P, Stinca S, Lacroix C: **The metabolic activity of gut microbiota in obese children is increased compared with normal-weight children and exhibits more exhaustive substrate utilization**. *Nutrition & Diabetes* 2011, **1**(7):e12.

7. Payne AN, Chassard C, Banz Y, Lacroix C: **The composition and metabolic activity of child gut microbiota demonstrate differential adaptation to varied nutrient loads in an in vitro model of colonic fermentation**. *FEMS Microbiol Ecol* 2012, **80**(3):608-623.

8. Cummings JH, Macfarlane GT: **The control and consequences of bacterial fermentation in the human colon**. *J Appl Bacteriol* 1991, **70**(6):443-459.

9. Florin T, Neale G, Gibson GR, Christl SU, Cummings JH: **Metabolism of dietary sulphate: Absorption and excretion in humans**. *Gut* 1991, **32**(7):766-773.

10. Gennari FJ, Weise WJ: **Acid-Base Disturbances in Gastrointestinal Disease**. *Clin J Am Soc Nephrol* 2008, **3**(6):1861.

11. Charney AN, Donowitz M: **Gastrointestinal Influences on Hydrogen Ion Balance**. In: *Acid-base disorders and their treatment.* Edited by Gennari FJ, Adrogué HJ, Galla JH, Madias NE: Taylor & Francis; 2005.

12. Davis GR, Morawski SG, Santa Ana CA, Fordtran JS: **Evaluation of chloride/bicarbonate. Exchange in the human colon in vivo**. *The Journal of Clinical Investigation* 1983, **71**(2):201-207.

13. Stephen AM, Haddad AC, Phillips SF: **Passage of carbohydrate into the colon: Direct measurements in humans**. *Gastroenterology* 1983, **85**(3):589-595.

14. Sung J, Kim S, Cabatbat JJT, Jang S, Jin YS, Jung GY, Chia N, Kim PJ: **Global metabolic interaction network of the human gut microbiota for context-specific community-scale analysis**. *Nat Commun* 2017, **8**.

15. Pudlo NA, Urs K, Kumar SS, German JB, Mills DA, Martens EC: **Symbiotic human gut bacteria with variable metabolic priorities for host mucosal glycans**. *mBio* 2015, **6**(6).

16. Duncan SH, Louis P, Thomson JM, Flint HJ: **The role of pH in determining the species composition of the human colonic microbiota**. *Environ Microbiol* 2009, **11**(8):2112-2122.

17. Batstone DJ, Keller J, Angelidaki I, Kalyuzhnyi SV, Pavlostathis SG, Rozzi A, Sanders WT, Siegrist H, Vavilin VA: **The IWA Anaerobic Digestion Model No 1 (ADM1)**. *Water Sci Technol* 2002, **45**(10):65-73.

18. Muñoz-Tamayo R, Giger-Reverdin S, Sauvant D: **Mechanistic modelling of in vitro fermentation and methane production by rumen microbiota**. *Anim Feed Sci Technol* 2016, **220**:1-21.

19. Baniya R, Upadhaya S, Khan J, Subedi SK, Mohammed TS, Ganatra BK, Bachuwa G: **Carbon dioxide versus air insufflation in gastric endoscopic submucosal dissection: A systematic review and meta-analysis of randomized controlled trials**. *Clinical Endoscopy* 2017, **50**(5):464-472.

20. Cotton PB, Williams CB: **Practical Gastrointestinal Endoscopy: The Fundamentals**, 6th ed edn: Wiley-Blackwell; 2008.

21. Swallow JH, Code CF: **Intestinal transmucosal fluxes of bicarbonate**. *The American journal of physiology* 1967, **212**(3):717-723.

22. Wang SP, Rubio LA, Duncan SH, Donachie GE, Holtrop G, Lo G, Farquharson FM, Wagner J, Parkhill J, Louis P *et al*: **Pivotal Roles for pH, Lactate, and Lactate-Utilizing Bacteria in the Stability of a Human Colonic Microbial Ecosystem**. *mSystems* 2020, **5**(5):e00645-00620.
