# Supplementary material for: Examination of hydrogen cross-feeders using a colonic microbiota model
Source: BMC Bioinformatics. 2021 Jan 6;22:3. doi: 10.1186/s12859-020-03923-6 (PMC7789523; doi:10.1186/s12859-020-03923-6)
Supplement: Supplementary file 2 — Additional file 2: Contains additional figures and a table of parameters of model fit. [file 12859_2020_3923_MOESM2_ESM.docx]

# Additional file 2

Additional figure 1. Model prediction compared with experimental data from Walker et al. [1] for continuous culture of a faecal microbial community on a medium containing 0.1% w/v peptide. In the left panel, measured metabolite concentrations are indicated by the coloured circles, solid lines indicate the model prediction using the Alpha parameter set, and dashed lines indicate the prediction using the Beta parameter set.

Additional figure 2. Model prediction compared with experimental data from Walker et al. [1] for continuous culture of a faecal microbial community on medium containing 0.6% w/v peptide. A pH shift from pH 5.5 to pH 6.5 was gradually enacted between days 6 and 8, as indicated by the dotted vertical lines. In the upper panel, measured SCFA concentrations are indicated by coloured circles, solid lines indicate the model prediction using the Alpha parameter set, and dashed lines indicate the prediction using the Beta parameter set.

Additional figure 3. A comparison of observed and modelled microbial relative abundances in each compartment after 10 days of continuous fermentation under each medium in the experiment of Payne et al. [2] with the obese child’s faecal material. Observed data was measured with qPCR and converted to microPop MFGs as described in Section 4, Additional file 1.

Additional figure 4. SCFA concentrations in the three fermenter compartments over the course of the 42-day experiment. This figure pertains to the experiment of Payne et al. [2] inoculated with faecal material from the normal-weight child, run in batch mode with high-energy (HE) medium for 2 days, then switched to continuous fermentation. During continuous fermentation, the model was run in four 10-day slots with differing media, in the following order: normal-energy (NE), HE, NE, low-energy (LE). The solid lines display the model prediction using the Alpha parameter set, whereas the dashed lines display the prediction using the Beta parameter set. The dotted vertical lines indicate changes between media.

Additional figure 5. A comparison of observed and modelled microbial relative abundances in each compartment after 10 days of continuous fermentation under each medium in the experiment of Payne et al. [2] with the normal weight child’s faecal material. Observed data was measured with qPCR and converted to microPop MFGs as described in Section 4, Additional file 1.

Additional figure 6. Model predictions (black lines) for SCFA production compared to experimental data (coloured lines represent the four subjects analysed) from Belenguer et al. [3] for a faecal culture at pH 5.5. The Alpha parameter set was used in this instance. Note that it was not possible to convert the data on the initial inoculum to microPop MFGs, thus three example faecal inocula derived from data in Walker et al. [1] are shown instead (black lines).

Additional figure 7. Model predictions (black lines) for SCFA production compared to experimental data (coloured lines represent the four subjects analysed) from Belenguer et al. [3] for a faecal culture at pH 5.5. The Beta parameter set was used in this instance. Note that it was not possible to convert the data on the initial inoculum to microPop MFGs, thus three example faecal inocula derived from data in Walker et al. [1] are shown instead (black lines).

Additional figure 8. Model predictions (black lines) for SCFA production compared to experimental data (coloured lines represent the four subjects analysed) from Belenguer et al. [3] for a faecal culture at pH 6. The Alpha parameter set was used in this instance. Note that it was not possible to convert the data on the initial inoculum to microPop MFGs, thus three example faecal inocula derived from data in Walker et al. [1] are shown instead (black lines).

Additional figure 9. Model predictions (black lines) for SCFA production compared to experimental data (coloured lines represent the four subjects analysed) from Belenguer et al. [3] for a faecal culture at pH 6. The Beta parameter set was used in this instance. Note that it was not possible to convert the data on the initial inoculum to microPop MFGs, thus three example faecal inocula derived from data in Walker et al. [1] are shown instead (black lines).

| **Additional table 1.** Mean bias (mM) of the model fits to SCFA datasets using either the Alpha or Beta parameter sets. The mean absolute error (mM) is shown in parentheses where different to the absolute value of the mean bias. | | | | | | |
| --- | --- | --- | --- | --- | --- | --- |
| **Walker et al. [1] dataset** | | | | | | |
|  | **Parameter set** | |  | |  | |
| **Figure 1** | **Alpha** | | **Beta** | |  | |
| Acetate | 10.6 | | 10.9 | |  | |
| Propionate | 2 | | 0.7 | |  | |
| Butyrate | -4.8 (5.2) | | -2.4 (2.8) | |  | |
| **Additional figure 1, Additional file 2** |  | |  | |  | |
| Acetate | 0.7 (2) | | 1.1 (2.3) | |  | |
| Propionate | 6.4 | | 4.6 | |  | |
| Butyrate | -3.9 (4.3) | | -0.6 (1.1) | |  | |
| Lactate | -2.8 | | -2.8 | |  | |
| Formate | -1.7 | | -1.7 | |  | |
| **Additional figure 2, Additional file 2** |  | |  | |  | |
| Acetate | 8.5 (9.2) | | 24.2 (24.9) | |  | |
| Propionate | 1.4 (2.5) | | -0.1 (2.6) | |  | |
| Butyrate | -3.4 (3.6) | | -11.2 (11.4) | |  | |
| **Figure 2** |  | |  | |  | |
| Acetate | <0.1 (4.1) | | 16.1 (16.6) | |  | |
| Propionate | 3 | | 1.4 (2.4) | |  | |
| Butyrate | <0.1 (2.6) | | -7.6 | |  | |
|  |  | |  | |  | |
| **Payne et al. [2] dataset** | | | | | | |
|  | **Compartment 1** | | **Compartment 2** | | **Compartment 3** | |
| **Figure 3** | **Alpha** | **Beta** | **Alpha** | **Beta** | **Alpha** | **Beta** |
| Total SCFAs | -27.5 (29.6) | -36.7 | 10.7 (20.1) | -3.6 (15.6) | -8.9 (20.7) | -23 (23.8) |
| Acetate | -37.7 | -48.4 | -16.4 | -26.6 | -31.7 | -41.4 |
| Propionate | -2.3 | -2.5 | 19.5 | 13.7 | 17.6 | 11.8 |
| Butyrate | 24.3 | 27.8 | 26.1 | 29.9 | 25.5 | 29.2 |
|  |  |  |  |  |  |  |
| **Additional figure 4, Additional file 2** |  |  |  |  |  |  |
| Total SCFAs | -31.5 | -24.8 | -5.1 (20.1) | 7.2 (16.5) | -24.9 (30.3) | -12.2 (17.2) |
| Acetate | -29.9 (31.2) | -18.9 | -33.8 | -16 | -36.7 | -18.4 |
| Propionate | -3.1 | -3.1 | 5.1 | 5.3 | 5.4 | 5.7 |
| Butyrate | 14.2 | 3.5 (6.1) | 41 | 28.9 | 40.5 | 28.3 |

# References

1. Walker AW, Duncan SH, Carol McWilliam Leitch E, Child MW, Flint HJ: **pH and peptide supply can radically alter bacterial populations and short-chain fatty acid ratios within microbial communities from the human colon**. *Appl Environ Microbiol* 2005, **71**(7):3692-3700.

2. Payne AN, Chassard C, Banz Y, Lacroix C: **The composition and metabolic activity of child gut microbiota demonstrate differential adaptation to varied nutrient loads in an in vitro model of colonic fermentation**. *FEMS Microbiol Ecol* 2012, **80**(3):608-623.

3. Belenguer A, Holtrop G, Duncan SH, Anderson SE, Calder AG, Flint HJ, Lobley GE: **Rates of production and utilization of lactate by microbial communities from the human colon**. *FEMS Microbiol Ecol* 2011, **77**(1):107-119.
